# Solid-state single-photon sources operating in the telecom wavelength range

**DOI:** 10.1515/nanoph-2024-0747

**Published:** 2025-05-05

**Authors:** Paweł Holewa, Andreas Reiserer, Tobias Heindel, Stefano Sanguinetti, Alexander Huck, Elizaveta Semenova

**Affiliations:** NanoPhoton – Center for Nanophotonics, DTU Electro, Department of Electrical and Photonics Engineering, Technical University of Denmark, Ørsteds Plads 343, DK-2800, Kongens Lyngby, Denmark; Technical University of Munich, TUM School of Natural Sciences, Physics Department and Munich Center for Quantum Science and Technology (MCQST), Garching, Germany; Institute of Solid State Physics, Technische Universität Berlin, Berlin, Germany; Department of Materials Science, Universitá di Milano-Bicocca, Milan, Italy; Center for Macroscopic Quantum States (bigQ), Department of Physics, Technical University of Denmark, DK-2800, Kongens Lyngby, Denmark

**Keywords:** quantum light sources, semiconductor quantum dots, color centers, rare-earth dopants, erbium, quantum communication

## Abstract

Solid-state quantum emitters operating in the telecom wavelength range are pivotal for the development of scalable quantum information processing technologies. In this review, we provide a comprehensive overview of the state-of-the-art solid-state emitters of single photons targeting quantum information processing in the discrete-variable regime and telecom wavelength range. We focus on quantum dots, color centers, and erbium ion dopants, detailing their synthesis methods and their applications. The review addresses the strategies for the integration of these quantum emitters into photonic devices alongside the associated challenges. We also discuss their applications in quantum technologies, examining current limitations, including performance constraints, decoherence, and scalability. Finally, we propose future directions for advancing photonic-based quantum technologies.

## 1 Introduction

Recent advances in quantum research have yielded groundbreaking developments in both fundamental quantum physics and nascent applications. Various hardware platforms are investigated in this context, each facilitating unique avenues for quantum information processing, sensing, and communication. The most prominent approaches include superconducting circuits, trapped ions, solid-state spin defects, quantum dots, cold atoms, and photonics. The photonic approach [1], [2] is unique. First, it enables long-distance data transmission over free-space and fiber-optical channels with minimal decoherence. This has opened an avenue for global quantum networks, quantum key distribution, and distributed quantum computing using remote systems. Second, nanophotonic structures can have tiny footprints, exhibit low loss, and be fabricated in large numbers by semiconductor foundries. In particular, low-loss platforms, such as Si or SiN, enable efficient on-chip data processing while benefiting from mature silicon fabrication technology and a readily available, well-developed photonics toolbox [3], [4], [5], [6], [7], [8]. This way, photonic devices may be scaled to multi-million qubit-based quantum computers [8]. While, so far, the operation of such platforms has been based on probabilistic single-photon sources, the integration of deterministic on-demand quantum light sources is promising for their up-scaling.

Research in quantum photonics can be divided into two distinct approaches, defined by the use of continuous or discrete variables as flying qubits and determined by the detection scheme, which resolves either the energy or the quadratures of the detected optical mode [9]. Continuous variable schemes leverage the amplitude and phase of the quantized electric field, while discrete variable schemes utilize streams of single photons that can be generated by various light emission processes. The latter can be further classified by the nature of photon generation: probabilistic sources, where photons are typically produced via nonlinear processes such as parametric down-conversion [8], and deterministic sources, which rely on optical transitions of atom-like systems. In this review, we will focus on the latter category, where the sources are operated by controlling the optical transitions of systems comprising two or more discrete energy levels.

An ideal source of single photons would consist of an atom in vacuum with an allowed dipolar transition, capable of emitting a stream of single photons on demand into a well-defined optical mode. The first experimental evidence of nonclassical light and signature of single-photon emission originates from a system consisting of a stream of single sodium atoms [10]. However, the approaches based on trapped atoms lack the practical functionality and mechanical stability to engineer a device with controllable and potentially tunable emission properties. Instead, placing the single-photon emitter in a solid-state medium offers substantial opportunities for engineering control over the photonic and electrical environment and, thus, the emission characteristics. However, it also introduces significant challenges with dissipation and dephasing arising from undesirable interactions of the single-photon emitter with the host crystal, such as phonons, magnetic noise from fluctuating electronic or nuclear spins, and charge noise associated, e.g., with crystal lattice imperfections or surface states.

Over the last decades, various approaches have been investigated in order to overcome these challenges for solid-state emitters [11], [12]. To this end, the emitters are placed into photonic structures, such as resonators or nanophotonic waveguides [13], [14]. In this setting, the Purcell effect [15] can enhance the emission into a single optical mode and reduce the lifetime to mitigate the detrimental impact of nonradiative transitions and emitter dephasing.

**Figure 1:**
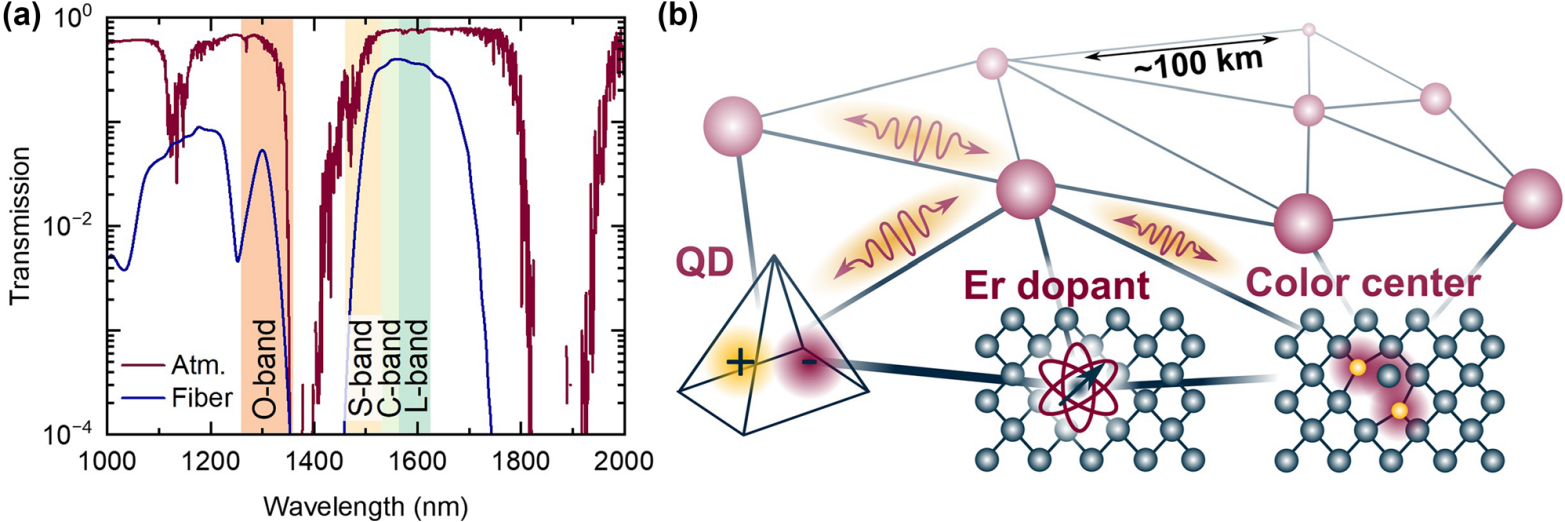
Photonic quantum networks operating at telecom. (a) Optical transmission of the atmosphere (dark red) and of a 20 km-long fused silica fiber (blue). Photons in the telecommunication bands (colors) exhibit low loss in both channels. (b) Scheme of a photonic quantum network with various quantum light sources: quantum dots (QDs), Er^3+^ dopants, and color centers such as the G-, T-, or W-center in silicon.

The field has progressed significantly, mostly exploring emitters in the visible and near-infrared (NIR) spectral ranges. In principle, photons at these wavelengths can be transmitted over global distances using satellites for direct atmospheric links [16]. However, long-haul connections can be implemented with reduced effort based on the low-loss characteristics of optical fiber networks and the well-established infrastructure of classical optical communication technology. This imposes a constraint on the operating wavelength of the emitter within the so-called telecom bands, specifically the 2nd telecommunication window with the O-band (1,260–1,360 nm wavelength), where the dispersion of silica is near-zero, and the 3rd telecommunication window, featuring S-, C- and L-bands (1,460–1,530 nm, 1,530–1,565 nm, and 1,565–1,615 nm, respectively), where the losses of fused silica optical fibers are minimal, ≤0.2 dB/km, as shown in Figure 1a. Although other materials have been explored for decades [17], lower losses have not been achieved so far. In addition to fiber-optical links, also free-space connections in the telecom bands allow for high transmission, as shown in Figure 1a.

For the above-mentioned reasons, solid-state quantum light sources operating in the telecom wavelength range have become a recent focus of research. This review will summarize the progress made in the last few years. We will present the fundamentals and latest advancements in epitaxial III-V quantum dots, rare-earth dopants in the solid state, and color centers in silicon and silicon carbide, as shown in Figure 1b.

The review is structured as follows: an introduction to the fundamentals of quantum light and the essential requirements for quantum light sources (Section 2); a comprehensive overview of each mentioned platform for telecom-wavelength quantum light sources, including fabrication methods of quantum emitters (Section 3); the engineering of quantum photonic devices based on these emitters (Section 4); an exploration of the applications of solid-state quantum light sources in quantum photonics (Section 5); and an analysis of the current challenges, limitations, and open questions within this field (Section 6). Finally, we discuss potential future research directions (Section 7).

## 2 Fundamentals of quantum light

Following the standard methodology, a single photon may be defined as a single excitation of a mode of the quantized electromagnetic field. The field mode is an orthonormal solution to Maxwell’s equations described by a set of parameters, including the center angular frequency *ω*, the spectral and temporal shape, the polarization, the transverse mode profile, and the propagation constant. Thus, an ideal source of single photons generates exactly one such excitation on demand. The quantized single excitation is the central figure of merit of a single-photon source and distinguishes it from excitations obtained from other sources of light, particularly thermal or coherent sources. However, depending on the specific use case and application of the single photons, the requirements on some of their parameters may be relaxed as it is the case for incoherent single-photon sources in the presence of spectral broadening effects.

Characterizing and benchmarking the quality of quantum light sources is routinely done in many laboratories using standard experimental techniques. These techniques have been thoroughly described in the literature [11], [12]; therefore, we will only summarize the most important aspects.

### 2.1 Measurement of photon statistics

The properties of the quantized electromagnetic field are fully described by the mode Wigner function or, equivalently, the density matrix $\hat{\rho }$ , where ${\hat{\rho }}_{1}=|1〉〈1|$ is the density matrix in the photon number basis of an ideal single photon. For a single mode, $\hat{\rho }$ can be obtained with a homodyne detector and reconstructed with a maximum likelihood algorithm [18], a process known as quantum state tomography. In practice, the homodyne detector requires a local oscillator that is a strong coherent reference field – in the optical domain delivered by a laser. Within the detection bandwidth of the homodyne detector, the local oscillator frequency has to match the frequency of the single photon. While a modified version of a homodyne detector has been implemented for an InAs/GaAs quantum dot (QD) single photon sources operating in NIR [19], the approach is challenging to realize for many experimental settings for two reasons. First, in case of low generation efficiency from the photon source, most of the time, the homodyne detector measures the vacuum state contribution, and second, spectral broadening effects and emission frequency fluctuations can be outside of the detector bandwidth.

Therefore, the most common approach for witnessing single-photon excitation in the light field and characterization of quantum light sources is done via the measurement of the normalized second-order correlation function *g* ^(2)^(*τ*) defined as

(1) $$g^{\left(2\right)}\left(\tau \right)=\frac{\left\langle {\hat{a}}^{†}\left(t\right){\hat{a}}^{†}\left(t+\tau \right)\hat{a}\left(t+\tau \right)\hat{a}\left(t\right)\right\rangle }{\left\langle {\hat{a}}^{†}\left(t\right)\hat{a}\left(t\right)\right\rangle \left\langle {\hat{a}}^{†}\left(t+\tau \right)\hat{a}\left(t+\tau \right)\right\rangle },$$

where $\hat{a}\left(t\right)$ and ${\hat{a}}^{†}\left(t\right)$ are the annihilation and creation operators of the single-mode field, averaging $\left\langle \cdot \right\rangle$ is done over time *t* and the term $\left\langle {\hat{a}}^{†}\left(t\right)\hat{a}\left(t\right)\right\rangle$ corresponds to the mean energy (photon number) of the field. To overcome timing resolution limitations and in the limit of low average photon counts, the *g* ^(2)^(*τ*) function can be efficiently measured with a Hanbury Brown and Twiss (HBT) setup, that is realized by splitting the mode 50/50 on a beam splitter and detecting the outputs with two single-photon sensitive detectors. The *g* ^(2)^(*τ*) function is then obtained by evaluating photon-click coincidences between the detectors and with respect to a relative shift of time by *τ*, and normalized by the average photon counts in both detectors. The detection bandwidth is only limited by the spectral responsivity of the detectors, and the measurement of *g* ^(2)^(*τ*) with an HBT setup is robust with respect to the photon source and setup efficiency, making the approach generally applicable to a wide range of photon emitters even in the presence of strong spectral broadening effects and very low detection efficiency.

Besides its robustness to photon loss, the *g* ^(2)^(*τ*) function has several useful properties that will be briefly summarized in the following. Applying the Cauchy–Schwarz inequality to Eq. (1), it can be shown that for any classical source of light *g* ^(2)^(*τ* = 0) ≥ 1. Therefore, the measurement of *g* ^(2)^(0) < 1 is a signature of nonclassical light. Regions with *g* ^(2)^(*τ*) = 1 correspond to photon statistics with a Poissonian distribution, while *g* ^(2)^(*τ*) > 1 corresponds to regions with increased probability of detecting two photons separated by *τ* and is thus termed photon bunching. Respectively, *g* ^(2)^(*τ*) < 1 refers to regions with a decreased probability of detecting two photons simultaneously, which is called photon antibunching. Finally, *g* ^(2)^(0) = 0 is characteristic for a single-photon excitation in the input optical mode. The quantity 1 − *g* ^(2)^(0) determines the conditional multiphoton probability. In parts of the literature, this is also called the “single-photon purity” and is an important parameter for many applications of single photons. The threshold *g* ^(2)^(0) = 0.5 is often used to identify single-photon emitters; however, in general, this criterion is insufficient as it only holds for the situation when the *n* ≥ 2 emitters contribute with the same average brightness [20]. Furthermore, analyzing the temporal evolution of *g* ^(2)^(*τ*) can be applied as a tool to study the internal level dynamics of quantum light emitters, while advanced analysis of coincidences between detection and no-detection events in the HBT setup can be used to conclusively reject models for quantum light emission [21].

### 2.2 Indistinguishability of single photons

Another important quantity is the indistinguishability of single photons, fundamentally linked to temporal and spectral broadening effects that may occur during the light emission process. The indistinguishability of photons can be determined by measuring the correlation function after interfering two photons either generated by different sources or consecutively by the same emitter when using a suited time delay, as shown in Box 1. To quantify the quality of the indistinguishability, one can perform two consecutive measurements – one in which the photons are indistinguishable and one in which they are made fully distinguishable, e.g., by rotating one of the polarizations to be orthogonal to the other. In a pulsed experiment, in which both photons arrive at the beam splitter in the same temporal mode, one defines the interference visibility *V* as:

(2) $$V=1-\frac{A_{\text{Co}}}{A_{\text{Cross}}},$$

where *A*_Co_ is the peak area in the co- and *A*_Cross_ the area in the cross-polarized two-photon interference (TPI) histograms for the central peak at zero delay. Perfectly indistinguishable photons will exhibit *V* = 1 at all delays, meaning they always leave the beam splitter in the same output port. This is called the Hong–Ou–Mandel effect. In contrast, fully distinguishable photons will not interfere (*A*_Co_ = *A*_Cross_) and yield a value of *V* = 0, corresponding to a random distribution of photons in the output ports.

If the emitted photons exhibit frequency fluctuations, this makes them partially distinguishable. In this case, the suppression of coincidences decreases with increasing delay time *τ* on a timescale set by the photon dephasing time *T*_2_. However, owing to the time-frequency uncertainty relation, the photons are still indistinguishable within short time windows, such that time-gated photon postselection can be used in some applications to achieve high postselected visibilities *V*_PS_ at the expense of reducing the efficiency or success rate of the experiment; see, e.g., Ref. [22]. Analyzing the postselected visibility also allows one to determine the coherence time of a photon source without pulsed excitation.

As an alternative to photon interference experiments, photon echo measurements – the optical analog to the well-known spin echo – have been used with single telecom emitters [23] to quantify their decoherence time. With several pulses, even dynamical decoupling [24] and correlation spectroscopy [25] on the optical transitions can be implemented, which allows for detailed investigations of the cause and timescale of spectral instability.

Box 1: Hong–Ou–Mandel experiment

**Figure:**
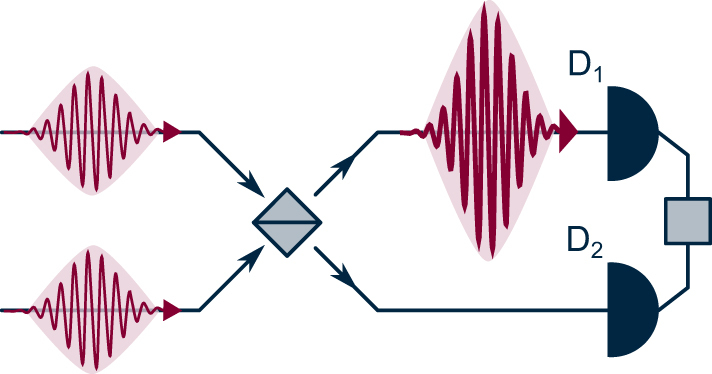

The degree of photon indistinguishability can be measured in a Hong–Ou–Mandel (HOM) experiment. Here, two photons (red curly arrows) impinge on separate input ports of a 50:50 beam splitter (gray), and detectors D_1_ and D_2_ are positioned at the output ports. There are three possible outcomes: both photons are detected by D_1_, both by D_2_, or one by D_1_ and the other by D_2_. The probabilities of these outcomes depend on the distinguishability of the photons, as summarized in the table below.

**Table:** 

Photons detected	Probability	
	Distinguishable	Indistinguishable
Both at D_1_	25 %	50 %
One at D_1_, other at D_2_	50 %	0 %
Both at D_2_	25 %	50 %

If the photons are indistinguishable in all degrees of freedom (frequency, spatiotemporal, and polarization modes), they will be detected in the same output port, i.e., both by D_1_ or both by D_2_.

## 3 Solid-state quantum light sources

Solid-state quantum emitters can be broadly categorized into two groups: First, QDs, which include many atoms and exhibit a large exciton confinement volume and thus a large polarizability; second, atomic-scale emitters, such as color centers and dopants, which are characterized by a much smaller spatial extent. In this section, we provide an overview of these types of solid-state quantum emitters operating in the telecom bands.

The first part of this section covers QDs and starts with introducing their general potential as quantum emitters. The discussion then shifts toward the challenges with QD growth to reach the emission at telecom, and the material systems explored to overcome them. The concept of fine structure splitting is introduced. The main growth approaches are introduced together with state-of-the-art results in respective fields: Stranski–Krastanov growth, droplet epitaxy and local droplet etching, site-controlled QDs, and nanowire QDs. This part is finished with a comment on the strain-induced quantum emitter in a 2D flake of MoTe_2_.

The second part describes the characteristics of color centers and rare-earth dopants. The section first focuses on color centers in silicon carbide and silicon, featuring G-, T-, and W-centers, and also describes methods to optimize their optical performance. Then, we will discuss erbium dopants and the consequences of their shielded electronic structure, as well as the influence of their host crystal on spin coherence and excited state lifetime. This part ends with a discussion of the spectral multiplexing of erbium emitters that is enabled by their narrow transition linewidths.

The final part of the section will discuss the similarities and differences between the various emitter systems covered in this review.

### 3.1 Quantum dots

III-V compound semiconductors offer great engineering flexibility for device fabrication, including band gap engineering, modification of the photonic environment, and formation of *p-i-n* structures with electrical contacts. Therefore, they constitute an ideal platform for the fabrication of quantum emitters in the solid state and provide the necessary tools to cope with the challenges introduced by the bulk environment.

Nanoscale three-dimensional epitaxial semiconductor heterostructures, or QDs, have demonstrated the ability to possess quantum-confined electrons and holes with a delta-like density of states whose energy, dynamics, and symmetry of the quantum state can be designed by the heterostructure composition, size, shape, and strain [26]. QDs are among the most advanced single-photon emitters for quantum information processing (QIP) purposes [27], [28], [29], [30], [31], [32], [33], in recent years witnessing a rapid development of QD-based technologies where a single QD is used as a source of photonic quantum states – single and indistinguishable photons [28], [29], [30], [34], [35], entangled photon pairs [34], [36], [37], [38], [39], [40], [41], also employing entanglement swapping [32], or multiphoton cluster states [42], depending on the application and quantum technology in scope. The field of epitaxial QD research spans approximately four decades. For a detailed exploration of the journey and milestones in this area, see the comprehensive review [43].

Despite many reported QD-based demonstrations in the field of QIP, the majority of them focus on the short ( < 1 µm) wavelength spectral range [27], [44], [45], [46], [47], [48], which – without frequency conversion [49] – impedes applications where photonic quantum states are transmitted over large distances using fiber networks (see Figure 1). This has stimulated significant research efforts dedicated to developing QD emitters tailored to the telecom wavelength ranges.

Recently, several reports tackling the main challenges of QD-based single-photon sources in the 3rd telecom window have appeared, proving that despite >20 years of research, it is a captivating subject that sparks significant interest in the community. Among others, noteworthy works include the demonstration of polarization-entangled photon pair generation by the biexciton–exciton cascade [39], coherent optical spin manipulation in InAs/GaAs QD grown on a metamorphic buffer [50] followed by the demonstration of entanglement between a single spin in an InAs/InP QD and a telecom photon, and showing the emission of photons from InAs/InP QDs with coherence times much longer than the Fourier limit via elastic scattering of excitation laser photons [51]. In addition, a fiber-coupled on-chip single-photon QD source in the telecom C-band has been implemented with a collection efficiency of up to 12.4 % into a single-mode fiber and high temporally postselected indistinguishability [52]. In the following, we will provide an overview of the progress in QDs operating at telecommunication wavelengths, highlighting advances in material systems and epitaxial techniques.

#### 3.1.1 Material systems for telecom quantum dots

The QDs are typically grown by molecular beam epitaxy (MBE) or metalorganic vapor phase epitaxy (MOVPE). There are three main material systems for QD emission in the 3rd telecom window. Two of them have been developed in parallel over the years: InAs/GaAs [53], [54], [55], [56] and InAs/InP [57], [58], [59], [60]. A third one has been introduced recently: GaSb/AlGaSb [61], [62].

##### 3.1.1.1 InAs/InP and GaSb/AlGaSb quantum dots

The advantage of InAs QDs in InP-based material systems is their natural coverage of the 3rd telecom window due to a lower lattice mismatch between a QD and the matrix, ∼ 3.2 %, in comparison to ∼ 7.2 % for InAs on GaAs. Single-photon emission with low multiphoton probability has been demonstrated [39], [63], [64], [65], [66], also under nonresonant excitation [64], rendering the InAs/InP QDs interesting for multiple applications in quantum technologies. Theoretical considerations have identified InAs/InP QDs as the best system to obtain low exciton fine structure splitting (FSS) for polarization-entangled photons in the 3rd telecom window [67].

Box 2 summarizes the biexciton–exciton cascade for the generation of polarization-entangled photon states and the importance of FSS within the Fourier-limited linewidth. The reason for lifting the degeneracy of the bright exciton states and the resulting nonzero FSS is the anisotropic electron–hole exchange interaction originating from the asymmetric QD confinement potential. Three factors contribute to the value of FSS:

1. The lattice mismatch between the dot and matrix materials enhances the magnitude of both intrinsic and shape-asymmetry FSS.
2. A stronger carrier confinement reduces the penetration of the wave functions into the matrix material, thus reducing intrinsic asymmetry effects.
3. Since the hole wave functions are more localized on the anion sites, having dot and matrix material with different anions will reduce the amplitude of hole wave functions at the interface, thus reducing the intrinsic FSS [67].

All three conditions for reducing the FSS energy can be met in InAs/InP QDs [67]. FSS measured for such QDs is as low as (12 ± 2) µeV [60]. These InAs QDs can also be placed in ternaries, e.g., InAlAs, or quaternaries, e.g., InAlGaAs, grown lattice-matched to InP. This, at the cost of an increase of the minimum achievable FSS, provides an additional degree of freedom in strain and bandgap engineering and shaping the barrier potential (carrier confinement regime) [60], [68], [69], [70].

Strain-free GaSb/AlGaSb heterostructures constitute the other promising material system for telecom QD emission, in which C-band QD operation has been demonstrated recently [61], [62].

##### 3.1.1.2 Metamorphic approach for InAs quantum dots on GaAs

QD emission at 1.3 µm and 1.55 µm in InAs/GaAs material system can be achieved by strain engineering. Targeting the reduction of built-in strain allows reducing the widening of the strain-induced InAs gap and thus red-shifts the QD emission [71]. This can be obtained by capping QDs with strained QWs (strain-reducing layer) [72]. A noteworthy demonstration showcased InAs QDs capped with a thin GaAsSb layer grown on a silicon substrate operating in the telecom C-band [73]. This work combines two techniques, strained capping layer and metamorphic growth.

The concept of the metamorphic technique is to engineer the lattice parameter of the matrix material hosting the QDs by employing a buffer layer with a material composition designed to promote plastic relaxation and localize resulting crystal defects (e.g., InGaAs layer with high In composition for the GaAs-based system). The technique was first demonstrated for single QDs in [53]. However, it remains challenging to reduce the high density of threading dislocations propagating through device layers that cannot be localized in the buffer layer and could cause charge noise due to the dangling bonds. Nevertheless, the metamorphic method gained significant attention a decade later [47], [55], [56], [74], [75], [76], [77] and enables single InAs/GaAs QD emission in telecom O- and C-bands. The indium composition in the metamorphic buffer can increase either linearly [74], [76], [78] or using a more complex, nonlinear In profile design [53]. The nonlinear grading profile allowed to minimize the thickness of the InGaAs metamorphic buffer on GaAs(001) down to 180 nm-thick layer for a full plastic relaxation, thus permitting its use within a planar cavity design [55].

High-quality lattice-relaxed metamorphic layers can be formed by the growth of InAs epilayers on (111)-oriented substrates, even at a large lattice mismatch, like InAs/GaAs(111)A [79], [80], InAs/Si(111), and InAs/GaSb(111)A [81]. This is related to the formation of misfit dislocations at the interface starting directly from the initial stages of the growth [79], being the {111} plane in a dislocation gliding plane in cubic crystals. The fast relaxation of the strain drives the system to grow two-dimensionally [80], thus allowing the fabrication of flat and thin metamorphic layers, e.g., InGaAlAs on GaAs(111)A substrates [82], [83]. Fully relaxed metamorphic InAlAs layers as thin as ∼40 nm are possible using (111)-oriented substrates [82]. High-quality single photon emitters have been realized by self-assembling InAs QDs on InAlGaAs using the (111)-oriented GaAs substrates, with the operation covering the telecom O-band [84], [85] and C-band [83], [86], [87].

#### 3.1.2 Quantum dot growth techniques

There are different techniques for synthesizing epitaxial QDs. Below, we will discuss two major QD self-assembling techniques, namely Stranski–Krastanov and droplet-based epitaxy, together with the inclusion of QDs in nanowires during growth. Finally, approaches for site-selective QD epitaxy will be presented.

##### 3.1.2.1 Stranski–Krastanov quantum dots

**Figure 2:**
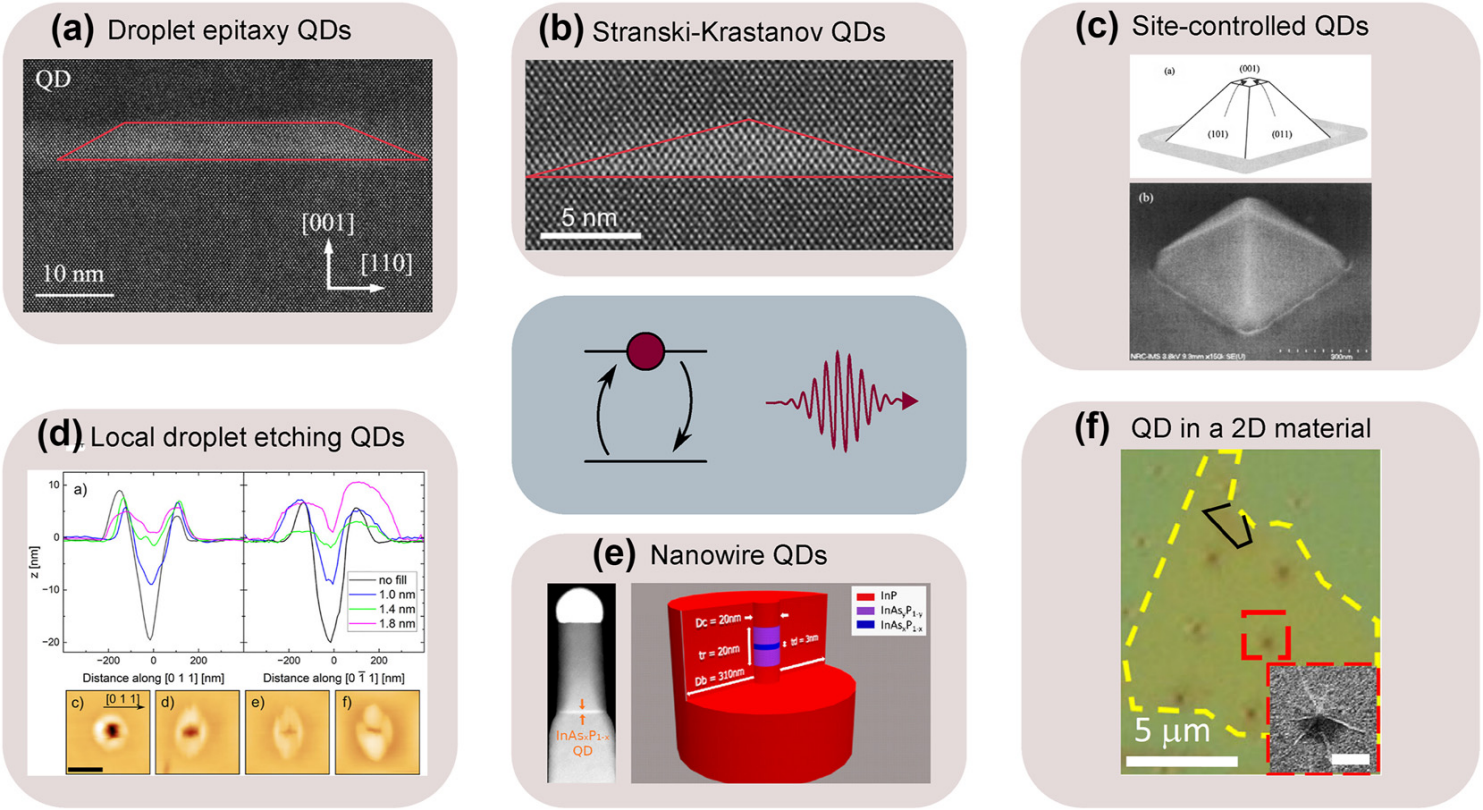
Examples of quantum dot growth techniques described in this review. (a) Droplet epitaxy QDs: atomic-scale scanning transmission electron microscopy (STEM) image in the cross-sectional geometry of InAs(P) (bright area) QDs in the InP matrix. The red line is included as a visual guide. (b) Stranski–Krastanov QDs: atomic-scale STEM image in a cross-sectional geometry of InAs (bright area) QDs in InP matrix. The red line is included as a visual guide. (c) Site-controlled QDs: schematic illustration and SEM image of an InAs QD formed at the apex of the InP pyramidal template. (d) Local droplet etching: cross-sectional atomic force microscopy profiles and plan-view images of droplet-etched holes in InAlAs filled with different amounts of InGaAs to form QDs. (e) Nanowire QDs: STEM image of InAsP QD embedded into InP nanowire. (f) QD in a 2D material: optical microscope image of a TMD monolayer flake outlined by yellow dashed lines (with a small bilayer region outlined in black) on a nano-pillar array. Inset: SEM image of a nano-pillar coated with monolayer TMD forming a QD. The scale bar of the inset is 500 nm. The central panel is a schematic illustration of the optical relaxation of an exciton in the two-level approximation. Panel (a) is adapted from Ref. [89], panel (d) from Ref. [90], the scheme in panel (e) from Ref. [91], and panel (f) is adapted from Ref. [92] under the Creative Commons Attribution 4.0 International License, Panel (b) and the STEM image in panel (e) are adapted from Refs. [57], [93], respectively, under the Creative Commons Attribution-NonCommercial-NoDerivatives 4.0 International License (CC-BY-NC-ND 4.0). Panel (c) is reprinted from Ref. [94] with the permission of AIP Publishing.

The most extensively explored QD systems to date are self-assembled QDs grown by the Stranski–Krastanov method [88] (Figure 2b). The strain-driven island nucleation mechanism [95], [96], [97] defines the parameters of the resulting QDs, determined by the lattice mismatch between the host material and the QD material. When the thickness *h* of the epitaxially deposited QD material exceeds an equilibrium value *h*_eq_, determined by the balance of surface and interface tension with the strain energy stored in the epilayer – typically ranging from one to a few monolayers (MLs) – it becomes energetically favorable for the epilayer to form 3D islands instead of continuing 2D pseudomorphic growth.

Box 2: Exciton fine structure splitting in quantum dots

**Figure:**
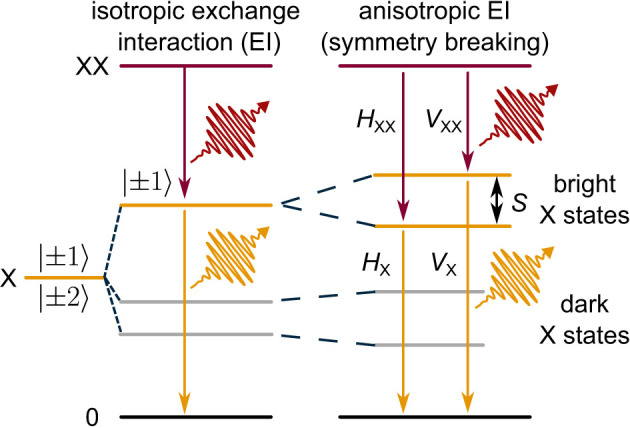


**XX-X cascade.** The radiative biexciton (XX) emission (red) leaves the dot occupied with an exciton (X). Due to the conservation of angular momentum, the X spin configuration is entangled with the polarization of the emitted XX photon. The polarization (*H* or *V*) of X photon (yellow) is predefined by the path taken during the XX recombination and leads to the emission of photons in the maximally entangled state

(3) $$|\Phi^{+}〉=1/\sqrt{2}\left(|H_{\text{XX}}H_{\text{X}}〉+|V_{\text{XX}}V_{\text{X}}〉\right),$$

as long as the exciton fine structure splitting (FSS) *S* is well below the homogeneous excitonic linewidth. Therefore, the degeneracy of bright exciton states (*S* → 0) is indispensable for erasing the “which-path” information from the XX–X cascade so that the polarization of XX and X photons cannot be inferred from their energy.

**Origin of FSS.** In a QD with an in-plane symmetric confining potential, the bright exciton states are degenerate, with |± 1⟩ being the eigenstates of the total angular momentum. However, the deviations from an in-plane cylindrical QD symmetry introduce anisotropic terms into the Hamiltonian describing the electron–hole exchange interaction (EI). This, in turn, introduces splitting *S* of the exciton bright states with new eigenstates $\left(|+1〉\pm |-1〉\right)/\sqrt{2}$ , generating linearly polarized photons for the XX–X cascade, |*H*_XX_⟩, |*H*_XX_⟩, |*V*_XX_⟩, |*V*_X_⟩.

This transition occurs because the three-dimensionality of the islands allows partial strain relaxation [96], [97]. The value of *h*_eq_ strongly depends on the lattice mismatch between the substrate and the epilayer and the epilayer–substrate interaction [96].

However, in actual growth conditions, the transition between 2D and 3D growth mode happens for the critical thickness *h*_c_, which is usually larger than *h*_eq_. After the transition, the additionally deposited material redistributes into partially relaxed 3D islands (the QDs), with a typical density of 10^10^–10^11^/cm^2^, on top of a 2D layer wetting the surface (the wetting layer) [95]. The critical thickness *h*_c_ is the value that limits the maximum achievable thickness of the wetting layer. *h*_c_ depends on the actual growth conditions and is usually larger than *h*_eq_. This effect can be explained as constraints imposed by growth kinetics preventing the system from achieving the equilibrium value. Thanks to the presence of such kinetic limitations in the QD self-assembly process, it is possible to tailor the properties of the resulting QDs, such as emission wavelength, by manipulating the growth conditions [98]. For example, changing the arsenic molecules in MBE growth from As_4_ to As_2_ enables a shape transition from dashes to dome-shaped QDs [99], inducing high in-plane symmetry of InAs/InP QDs, demonstrated also for single QDs [100].

Achieving a low surface density of QDs in the order of 1 per µm^2^ is critical to ensure that each processed device comprises a single QD, critical for applications in the quantum domain. Operating in the “near-critical” regime (*h*_eq_ < *h* < *h*_c_) allows for almost independent control over both the surface density and size of QDs [57], which otherwise cannot be decoupled [101]. This method allows achieving bright InAs/InP QDs single-photon sources with a low multiphoton probability in the telecom C-band using nonresonant and quasi-resonant excitation, as well as on-demand generation of indistinguishable photons [102], [103]. The near-critical method also applies to metamorphic InAs/InGaAs/GaAs systems [104].

Alternative approaches to reduce the surface density is the QD ripening [105], [106], [107]. This yields high-quality single-photon emission [107], also for QDs of high in-plane symmetry [64]. The double capping technique [108] results primarily in lowering the QD height but also in surface density reduction with high-quality single-photon emission obtained under nonresonant [109] and quasi-resonant excitation [63], [110].

##### 3.1.2.2 Droplet epitaxy quantum dots

**General description.** A different growth mode for the self-assembly of III-V QDs is droplet epitaxy [60], [62], [87], [89], [111], [112], [113] (Figure 2a). The QD nucleation in this approach does not rely on lattice mismatch between the epilayer and the substrate, as opposed to a strain-driven Stranski–Krastanov growth. Here, group III metal droplets (e.g., In, Al, or Ga) are initially self-assembled on the substrate. Droplet epitaxy can be performed on any crystalline substrate, from III–V semiconductors [60], [62], [114] to Si [115]. The droplet density can be controlled via the substrate temperature and group III flux [116], [117], [118], [119]. The achievable droplet density can be tuned from 10^6^ to 10^12^ cm^-2^. The droplet volume is controlled by the total amount of metal deposited. The droplets are then annealed in the group V (As, P) flux. During the annealing step, the metal droplet is transformed into a crystalline nanoisland [120], [121]. By changing the annealing conditions (substrate temperature and group V flux), as well as the postcrystallization annealing [89], it is possible to tune to the nanoisland shape and thus the QD electronic states [122], [123], [124]. The droplet deposition and annealing are independent, enabling precise control over QD array properties [111]. This makes droplet epitaxy a powerful method for tailoring the QD shape, size, and density, and thus their optical properties.

**Droplet epitaxy at telecom.** The fabrication of InAs/InP QDs by droplet epitaxy in an MOVPE environment proceeds as follows: an indium flux is supplied to the InP substrate, forming metal droplets in random positions. The droplets are annealed under arsine flux, leading to the droplet crystallization and formation of InAs QDs. In this system, an emission wavelength in the telecom C-band can be naturally achieved [39], [60], [65], [125] with the surface density of QDs on the level of 10^8^ cm^−2^ [89], [125].

InAs droplet epitaxy QDs are typically in-plane symmetric because the crystallization process is less sensitive to indium surface diffusion, which is an anisotropic process. A high level of symmetry can be achieved on standard (001)-oriented InP, as demonstrated, e.g., in Refs. [60], [89]. By a proper choice of substrate orientation, like (111)-oriented InP, extremely in-plane symmetric QDs can be achieved [113]. This results in significantly lower exciton FSS compared to Stranski–Krastanov QDs, rendering droplet epitaxy QDs particularly interesting as sources of polarization-entangled photon pairs [60]. The high symmetry enabled demonstrations of quantum entanglement under electric excitation [39] and quantum state teleportation [65]. Highly symmetric droplet epitaxy InAs/InAlGaAs QDs have been reported, with single photon emission and low FSS (< 20 µeV) both in the O-band [84], [85] and C-band [87]. For details on the droplet epitaxy and related QD symmetrization, see Refs. [111], [118], [123].

##### 3.1.2.3 Local droplet etching quantum dots

Annealing of the metallic droplet under moderately low V group flux can lead to a local droplet etching of the underlying III–V material instead of droplet crystallization [126]. The result of this process is a formation of highly symmetric pits by etching the material beneath the droplet, accompanied by the redistribution of the etched material and the droplet material around the pit’s circumference [126]. The pits are then filled with a lattice-matched material to the host matrix, forming QDs [111]. This method is particularly powerful because it mitigates symmetry-breaking effects such as strain and shape anisotropy, as well as compositional gradients resulting in ultra-small FSS [111]. For the telecom wavelength regime, this method was recently demonstrated by filling the holes in InAlAs by InGaAs in the InP system, covering O- and C-band emission wavelengths [90] (Figure 2d) and in GaSb/GaAlSb, with QDs emitting in the telecom L-band with a very low FSS, (12.0 ± 0.5) μeV [61], [62].

##### 3.1.2.4 Site-controlled quantum dots

In the methods of QD growth that we have discussed so far, the position of each QD is random. However, it is beneficial to have spatial control of the QD nucleation to position a quantum emitter at a specific location, e.g., within a photonic cavity, with high accuracy. There are various approaches to site control QD nucleation that include etching the holes either lithographically [127], [128], [129], [130], [131] or assisted by atomic force microscopy [132], growth through a dielectric mask [133], as well as strain engineering in underlying layers [134], [135], [136], [137]. For telecom wavelengths, a demonstration used InAs QDs on top of InP pyramids grown through a dielectric mask [133], [138]. Tailoring growth parameters allows controlling the geometry of pyramids [138] (Figure 2c) and the QDs’ polarization properties [133].

##### 3.1.2.5 Nanowire quantum dots

Nanowires are 1D structures that are typically grown using vapor–liquid–solid (VLS) [139] or selective-area epitaxy (SAE) [140], allowing precise positioning of nanostructures. The diameter of the nanowire could vary from tens to hundreds of nanometers. To form a QD within a nanowire, either a segment of a different crystal structure (wurtzite and zinc-blende) [141] or a layer of a different material (such as InAs(P) in InP) [142] can be introduced. This insertion confines charge carriers in all three dimensions, effectively acting as a 0D object due to the small diameter of a nanowire. Enhancing or suppressing epitaxial growth in lateral and axial directions is possible by manipulating the growth parameters. This enables the formation of structures such as quantum dots as well as the encapsulation of a nanowire core within a shell, thereby mitigating the impact of surface states on the QD properties [91].

The emission in the telecom range has been reported for InAs QDs in InP nanowires [93], [142], [143]. Nanowires can function as waveguides, enhancing the collection efficiency of emitted light [142]. A recent demonstration involved position-controlled QD emitters embedded in nanowires, operating at 1.31 µm with a collection efficiency of 27.6 % and exhibiting a very low probability of multiphoton emission even at elevated temperatures [91]. Other demonstrations feature single-photon generation with low multiphoton probability, moderate photon indistinguishability, and the first-lens source efficiency of 28 % [144], coherent control of excitons from a resonantly driven nanowire [145], and the on-demand generation of polarization-entangled photon pairs due to the naturally low FSS of 4.6 µeV [146] – all in the telecom O-band.

##### 3.1.2.6 QDs in 2D materials

Another type of QDs can be formed in 2D materials. As far as single-photon emission is concerned, monolayer transition metal dichalcogenides (TMDs) are considered a very promising platform because of their rich range of possibilities for hetero-integration in photonic devices. In addition, for a 2D system, due to its atomic-scale thickness and strong in-plane bonds, the emitted photons can be easily extracted, facilitating the emitter integration with various optical components, such as waveguides and optical fibers. A fundamental property of these systems is related to the deterministic generation of quantum dots in such monolayer TMDs in the form of potential pockets for the excitons by strain seeding. Local strain leads to a local reduction in the band gap. Such local potential minima may capture free excitons and direct them into emitting defect-bound states. For a general overview of single-photon emission by 2D materials, see recent reviews by Esmann et al. [12] and Zhang et al. [147].

Generally, single-photon emission from TMD is restricted to the visible and near-infrared wavelength range [12], and extending the emission to the telecom range is an ongoing effort. The emission in the C-band has been reported only once for TMD multilayers of MoTe_2_ [92]. The telecommunication single-photon emission at 1.55 µm was demonstrated for excitons confined in this material using the strain induced by nano-pillar arrays. These emitters exhibited photon antibunching at cryogenic temperatures with *g* ^(2)^(0) = (0.155 ± 0.009) when time-gated at 200 ns [92].

### 3.2 Color centers

In the preceding section, it was summarized how *artificial* atoms in the form of quantum dots can be used as efficient sources of nonclassical light. In the following, we will consider instead real atoms that are embedded as impurities or dopants in the crystal lattice of another material. There is a very large variety of such defects that can exhibit atom-like properties; in case they can be used for light emission, they are often referred to as luminescent defects or color centers. See Refs. [148], [149] for recent review articles.

Compared to quantum dots, the color centers exhibit a key difference: Their emission wavelength is not tunable over a large range but occurs within a comparably narrow frequency regime. In a perfect crystal without any strain inhomogeneities, the emission of all color centers of a certain type would be identical. However, in typical high-purity materials, a residual inhomogeneous broadening is observed that ranges from tens of MHz [150] to hundreds of GHz, depending on the emitter and host properties [151].

The first color centers that were studied on a level of individual emitters were the Nitrogen-Vacancy (NV) centers in diamond, which can be used as single-photon sources up to room temperature. When operating at cryogenic temperature, a fraction of the light emitted into the zero-phonon line is phase-coherent and can be used for quantum networking, which has allowed many pioneering experiments following the first demonstration of remote entanglement [22].

From a general perspective, the emission wavelength of a color center will depend on the spatial confinement of the electrons. For emitters in diamond, including the NV center and others based on the fourth column of the periodic table [151], this entails an emission in the visible spectrum, e.g., at 637 nm for the NV center. While this wavelength exhibits strong absorption in optical fibers, efficient conversion to the telecommunications bands can be used for long-distance connections [152]. However, this comes at the price of reduced efficiencies and added noise. Thus, an intense search for color centers that emit directly at telecommunications wavelengths has been started in the last few years. In addition, other host materials have come into focus, as diamond is difficult to grow on a wafer scale, and doping and nanofabrication are more difficult compared to other established semiconductors, such as silicon and silicon carbide.

#### 3.2.1 Color centers in silicon carbide and silicon

Many recent experiments have investigated color centers in silicon carbide. Several vacancy-related emitters have been identified that emit in the near-infrared spectral region [148]. To further push this to the telecommunications bands,NV [153] and transition-metal impurities have been studied, whose transitions fall into the O-band [154], [155]. While coherent light emission requires a cryogenic operation, incoherent bright single-photon sources can be implemented even at room temperature, enabling up to MHz detection rates [153], [156].

Remarkably, many color centers in SiC can also host coherent spins, which may open the door for spin-photon entanglement and quantum networking experiments [157]. As an example in the O-band, the first measurements on ensembles of vanadium vacancies resolved the different spin transitions using optically detected magnetic resonance spectroscopy [158]. The spin lifetime of ≲ 1 μs at 3.3 K is expected to be strongly improved upon cooling to millikelvin temperatures.

To enable two-photon interference experiments, the emitted photons should exhibit the same or close-by frequencies. This can be achieved in low-strain host crystals. Remarkably, even the presence of different isotopes of Si and C in SiC – both on the few-per-cent level – can lead to a significant inhomogeneous broadening of the transitions. Ref. [159] summarizes the effects of the host isotopes on the optical spectra of semiconductors. Spectral broadening can be dramatically reduced in isotopically purified material [160]. As a recent example, a residual fluctuation of the transition frequency of several single emitters in the same crystal as low as 0.1 GHz has been reported [161].

This makes these emitters promising for future experiments on two-photon quantum interference, or even to explore the collective coupling of multiple single-photon sources [14]. To this end, the emitters should be integrated into suited resonators or nanophotonic structures to overcome the limitations posed by nonradiative and phonon-sideband transitions. This will be described in more detail in Section 4.

**Figure 3:**
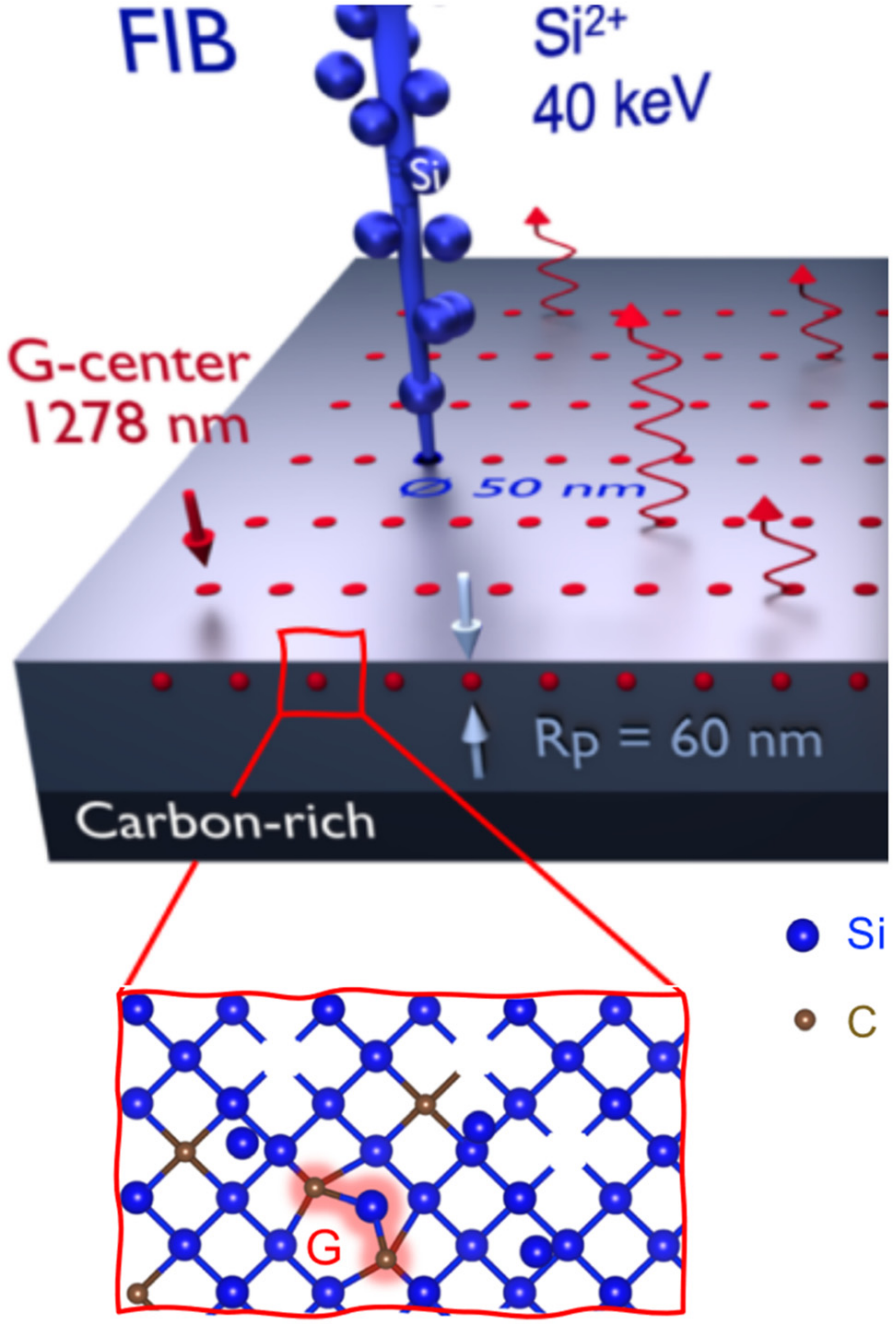
Creation of color centers by local implantation. A focused ion beam (FIB) is used to implant Si atoms (blue) at well-defined positions (red) into a carbon-rich layer of crystalline Si. Inset: The created interstitial Si atoms (blue) can form G-centers (red) together with a pair of substitutional C atoms (brown). At low Si implantation doses, single G-centers are produced probabilistically that can be used as single-photon sources in the telecom O-band. Figure adapted from Hollenbach et al. under a CC-BY license [169].

While SiC photonics is actively developed [162], embedding color centers in Si would offer the advantage that a well-developed photonics toolbox is readily available [3], [4], [5], [6], [7], [8], [163]. However, silicon has a much smaller bandgap than diamond and SiC. Thus, the effects of emitter ionization, two-photon absorption, and nonradiative decay need consideration in this material. Albeit many color centers have been known for decades [164] to emit light at telecommunication wavelengths, individual emitters in silicon have only come into focus very recently. First studies investigated single defects called W-centers [165], G-centers [166], [167], and T-centers [168], as well as other emitters of yet-unknown structure [166]. All of these color centers are associated with carbon impurities in silicon and thus occur naturally in bulk material. Alternatively, they can be fabricated by nonequilibrium techniques such as implantation and annealing. Recently, even spatially selective emitter formation by implantation with a focused ion beam has been shown, achieving a resolution of approximately 0.1 µm [169], as shown in Figure 3. Recently, this approach has been extended by deterministically fabricating CBG cavities centered around W-centers fabricated by implantation into an SOI sample [170]. Other experiments demonstrated a local activation of color centers by pulsed laser annealing [171], [172].

#### 3.2.2 Optimizing the properties of color centers

In addition to spectral diffusion, the performance of single-photon sources based on color centers can also be affected by optical pumping to a state that does not couple to the repeated excitation laser pulses, such that photon emission is inhibited for some time. This “blinking” can be caused by metastable internal states of the defect, in particular, charge states that can be very long-lived. To avoid this, several techniques can be used to stabilize the desired charge state using tailored doping profiles or electrodes [151]. Alternatively, it can be recovered periodically by charge-reset pulses that drive transitions out of the metastable state.

Even if the emitter charge state itself is stable, charge traps in its surroundings can also be detrimental: If they change state, the Stark shift can lead to sudden jumps of the emitter frequency. For traps in the proximity of the emitter, as typically encountered at close-by interfaces, the resulting spectral diffusion typically exceeds the radiative lifetime by orders of magnitude. To eliminate this obstacle, the charge state of surrounding traps can be stabilized to some degree by integrating the emitter into large static electric fields [65]. This approach can also allow tuning the emission frequency such that two emitters become resonant. While these techniques have been pioneered with color centers in the visible spectrum, the first experiments with telecom O-band emitters in silicon have been reported recently [173].

As an alternative, defects that belong to certain symmetry classes can be used that experience no Stark shift to first order [174]. Encouraging results have been obtained with group-IV defects in diamond that emit at visible frequencies [151], but so far, no color center with such symmetry properties has been reported at telecom wavelengths. However, in particular, when it comes to nanophotonic integration, this seems to be mandatory, or at least highly advantageous, for quantum information protocols that rely on photon interference and thus spectral stability of the emitters [163], [175]. In addition, quantum networking over large distances would require emitters in the telecom C- or L-bands, where losses in optical fibers are minimal. However, the color centers reported above only range up to the O-band, where the transmission after 100 km is about thousandfold smaller, such that again low-noise photon conversion would be required for practical rates. Finally, for distributed quantum protocols, the emitters should also exhibit a long-lived spin that can be entangled with the polarization or emission time of a photon.

In SiC, long-lived spins and coherent optical emission are expected for several emitters [157]. In contrast, in silicon, this requirement is only fulfilled by the T-center [168], which exhibits an electronic and nuclear spin with lifetimes beyond a millisecond or even a second, respectively, in isotopically purified ^28^Si [160]. With this, one expects very long coherence under dynamical decoupling that can eliminate remaining couplings to paramagnetic impurities, residual nuclear spins, and other quasi-static fluctuations [151]. Unfortunately, the 1 µs optical lifetime of the T-center is relatively long, and it exhibits significant nonradiative decay. Thus, even in nanophotonic cavities, the achieved two-photon interference contrast currently does not allow for high-fidelity remote entanglement [176].

For the reasons mentioned above, the quest is still open to find color centers with improved properties. Recent advances in ab initio calculations [177] and the availability of sufficient computing power have enabled high-throughput numerical calculations of thousands of point defects in various semiconductors, including diamond, silicon carbide, and silicon [178]. Still, experiments will be required to explore whether these emitters exhibit improved properties.

### 3.3 Rare-earth dopants

Similar to color centers, also rare-earth dopant atoms in their triply ionized charge state can be used as quantum light sources. Compared to all other solid-state emitters, they have quite unique properties, as their 4f electrons are enclosed by fully occupied 5s and 5p electronic shells. These shells act like a Faraday cage and thereby protect the 4f levels from the electric fields of the surrounding atoms of the host crystal. Thus, the spectra obtained for optical transitions between 4f levels are largely independent of the host material [179]. The surrounding crystal only acts as a perturbation to the Hamiltonian of the free rare-earth ion, splitting the 4f energy eigenstates that would be degenerate in free space into so-called crystal field levels [180]. Phonons can induce fast transitions between these levels. Thus, using rare-earth dopants for quantum applications requires cooling to cryogenic temperature – 4K or even less – such that only the lowest crystal field level is occupied. In this situation, rare-earth emitters can serve as efficient two-level systems to generate single photons [181]. As the optical transition energy exceeds the phonon energies of most crystals by an order of magnitude or even more, nonradiative transitions are negligible in all host crystals (with only very few exceptions, see, e.g., Ref. [180]).

In addition, rare-earth dopants can also exhibit very long-lived spin levels that can serve as qubits with outstanding coherence properties in host crystals with a low density of magnetic moments. As an example, under optimized magnetic fields, the coherence of hyperfine ground states can exceed several hours [182], and optical transitions can exhibit millisecond coherence times [183], exceeding other solid-state emitters by several orders of magnitude. This has allowed for many pioneering experiments on solid-state quantum memories [184]. Still, experiments with single emitters were impeded by the long lifetime of the optically excited states, which is often on the order of several milliseconds. This has only changed recently, enabled by the integration of the emitters into optical resonators [181], [185], [186]. With this, rare-earth dopants have also emerged as a promising platform for the generation of coherent photons that are entangled with long-lived quantum memories and may be considered a solid-state alternative to quantum networking experiments with atoms trapped in vacuum [187].

#### 3.3.1 Erbium dopants

For long-distance transmission, the rare-earth element erbium is of particular relevance, as it emits photons in the telecom C-band, where the loss in optical fibers is minimal. Thus, erbium has received considerable attention in recent years and will be the focus of this section.

In principle, erbium can be integrated during growth or via implantation [188], [189] in a wide variety of host materials, with optical transitions between the lowest crystal-field levels in a typical range between 1,530 nm and 1,540 nm. A well-known example is erbium-doped glass fibers, which allow for the implementation of fiber lasers and amplifiers that form the technological backbone of today’s internet. For photon sources, however, crystalline hosts are preferred: First, they exhibit a much lower density of perturbations (such as impurities or two-level systems) and thus better coherence. Second, they give access to optical transitions with a narrow inhomogeneous broadening, with typical values below 1 GHz that can be easily bridged by optical modulators. Thus, every erbium dopant can be optically interfaced with any other one (in the same host material) – even without tuning procedures that are required for other solid-state emitters. In isotopically pure crystals, inhomogeneous linewidths as low as 16 MHZ have been observed [190].

Also the spin transitions of Er dopants can be narrow, with a typical inhomogeneous broadening in the range of a few MHZ for the electronic spin. Even at concentrations of only a few ppm, this value is limited by erbium–erbium interactions that cannot be decoupled because of the anisotropy of the magnetic moment of the dopants [191]. Thus, lower linewidths require ultrapure crystals [192], or the use of hyperfine transitions of the only erbium isotope with nuclear spin, ^167^Er [193]. Alternatively, one can reduce the spin- and/or optical linewidth by applying specific magnetic fields, where transition frequencies can be insensitive to first order [194], [195]. But even without this technique, the spin lifetime can exceed many seconds for both electronic spins [192] and hyperfine levels [193], and the spin echo coherence time can reach 23 ms and 1.3 s, respectively, even without dynamical decoupling. Thus, the ground-state coherence can be considered long enough for all applications described in this review.

#### 3.3.2 Choice of the host crystal and integration techniques

In principle, almost any host material can be used to implement single-photon sources based on erbium emitters. However, the coherence time of both ground-state and optical transitions will strongly depend on the choice of the host. The main considerations in this context will be summarized in the following.

The first important aspect is the symmetry of the host crystal and the erbium site. To ensure that each dopant emits approximately at the same frequency, erbium should integrate in a single site, or at least only in a few. This site should exhibit a low symmetry, such that all crystal field levels are split; this splitting should be large, such that only the lowest level is populated at a temperature that is conveniently accessible with ^4^He cryostats. In addition, the site should exhibit a large oscillator strength of the telecom transitions to enable fast operations. Furthermore, as erbium has a strong magnetic moment, one needs ultrapure crystals with a low density of paramagnetic impurities unless operation at large magnetic fields or ultra-low temperatures is used to freeze the electronic spins surrounding (and including) the erbium emitter. For the same reason, crystals with a low density of nuclear spins, or at least with low nuclear magnetic moments, are advantageous. Finally, one will aim for a site in which erbium has a small or even vanishing first-order Stark coefficient to make the optical transitions insensitive to electric field noise in the host [174].

Typical material choices that fulfill all or at least most of the above requirements and have been used for the generation of single photons are Y_2_SiO_5_ [23], [24], [181], YVO_4_ [185], Y_2_O_3_ [199], CaWO_4_ [200], LiNbO_3_ [201], and silicon [196], [197]. An overview of the properties of erbium in these host materials is given in Table 1.

**Table 1:** Survey of the host materials in which single erbium dopants have been used to generate single photons. Extended and adapted from [189].

	Si	Y_2_SiO_5_	Y_2_O_3_	CaWO_4_	LiNbO_3_
Emission wavelength [nm]	1,537.76	1,536.14	1,532.2	1,531.52	1,529.2
Optical lifetime in bulk [ms]	0.14	11.4	7.3	6.3	2
Inhomogeneous linewidth [GHz]	0.4	0.2	0.4	1	30
Homogeneous linewidth in bulk [kHz]	< 10	0.07	0.6	2	3
Crystal field splitting Z_2_ [THz]	2.6	1.9	1.2	0.6	1.9
Crystal field splitting Y_2_ [THz]	2.4	1.2	1	0.25	1.9
Debye temperature [K]	640	580	230	267	503
Single-spin lifetime [s]	0.4 @ 3 K	20 @ 0.5 K	–	0.4 @ 0.5 K	–
Single-spin Hahn echo time [ms]	0.05 @ 3 K	0.002 @ 0.5 K	–	0.04 @ 0.5 K	–
Single emitter reference	[196], [197]	[23], [198]	[199]	[200]	[201], [202]

To achieve fast emission in the mentioned experiments, optical resonators have been implemented by focused ion beam milling [185], heterogeneous integration [181], [200], [201], and using dielectric mirrors fabricated by laser ablation [23], [199]. Remarkably, in silicon standard processes of the semiconductor industry can be used for foundry-based device fabrication while preserving narrow optical transitions [203]. This may pave the way for the fabrication of quantum light sources on a large scale.

In all of these experiments, erbium was integrated at random positions in the resonator. Thus, the maximum Purcell enhancement was only achieved for a small subset of the emitters. This limitation can be overcome in the future by masked implantation. Controlling the acceleration voltage enables position control with a typical straggling of tens of nanometers, well within a single maximum of the intracavity field. Subsequent annealing is required to heal the implantation damage and integrate the emitters into the lattice [188], [189]. There, a trade-off between the emitter localization and the crystal quality may be encountered, which thus requires a thorough optimization of the annealing conditions.

#### 3.3.3 Spectral multiplexing

**Figure 4:**
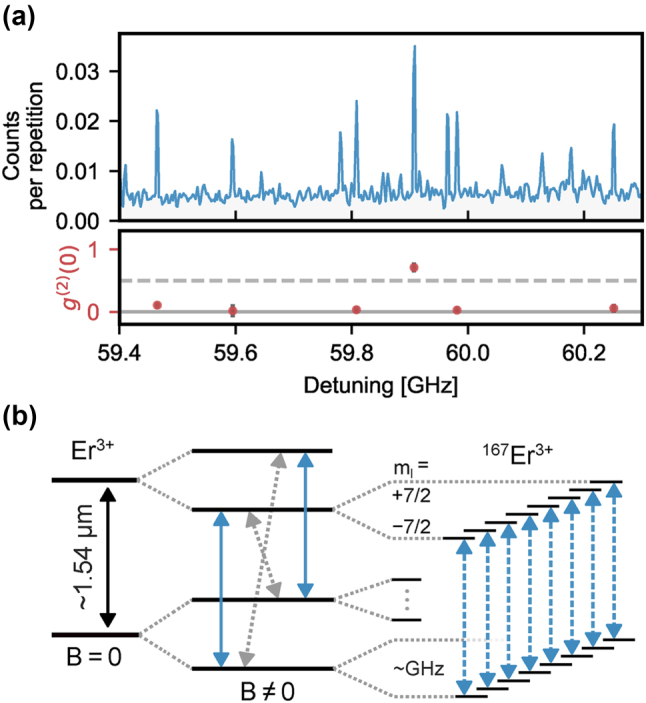
Spectral selection and individual control of multiple erbium emitters. (a) Spectrally multiplexed single-photon source in a Fabry–Perot cavity. A laser with precisely controlled frequency is used for pulsed excitation of the emitters. The fluorescence after the pulses exhibits distinct peaks (top), most of which correspond to single emitters, as evidenced by the antibunching in the dark-count corrected photon correlation function (bottom). As the spectral diffusion linewidth is much narrower than the inhomogeneous distribution, hundreds of emitters can be spectrally resolved and multiplexed. Adapted from [24]. (b) Level scheme of erbium emitters. Each dopant is characterized by an effective electronic spin 1/2, with an anisotropic splitting that depends on the applied magnetic field direction. The isotope ^167^Er further exhibits a 7/2-nuclear spin that can achieve second-long coherence when a large magnetic field freezes the electronic spins in their ground state [193].

While the long lifetime of the optically excited state of erbium emitters can impede the rates of photon generation, it also provides novel possibilities. In particular, it is feasible to integrate many single-photon emitters into the same optical resonator while still individually addressing them using narrowband excitation. The reason is that the optical linewidths of the dopants, often below 10 MHz, can be much narrower than the inhomogeneous distribution of the transition frequencies that is on the order of 1 GHz in typical hosts with a rather homogeneous strain. Thus, at a low enough concentration, each dopant will emit at a slightly different frequency that can be easily resolved in spectroscopy. Excitation with a narrow-linewidth laser thus enables spectral selection and individual control of many emitters, as shown in Figure 4.

When the cavity resonance can be actively tuned, the total number of emitters may easily exceed several hundred. As an example, 360 emitters with a Purcell enhancement >35 have been reported in [24], as shown in Figure 4a. This number will eventually be limited by the ratio of the inhomogeneous distribution and the spectral diffusion of the emitters. The latter can be lower than 200 kHz [23], [200], while the former can be broadened on purpose to many GHz by introducing strain, e.g., by codoping [24].

A key property of the erbium emitters is that each of them provides an electronic spin with long coherence, and – when the isotope ^167^Er is used – an additional nuclear spin that can both be used for quantum information storage on the timescale of milliseconds [192] or even seconds [193]. Together with their C-band emission, this makes erbium emitters a prime candidate for long-distance quantum networking, which will be further discussed in Section 7.

## 4 Engineering of quantum devices operating in the telecom wavelength range

Placing quantum emitters in a solid-state matrix poses two challenges that are common to all solid-state platforms. The first is the refractive index contrast between the host solid-state material and its surroundings. In high-index materials, total internal reflection at the interface poses a challenge to the photon extraction efficiency, with typical values of ∼ 1 %. The second challenge originates from interactions between the charge carriers confined in the emitter and their solid-state environment that can induce dephasing and thus limit the emitter’s applicability in quantum photonic devices.

Therefore, engineering of the local photonic environment is required for all solid-state emitters to address these challenges by (i) improving the photon extraction efficiency and (ii) increasing the radiative transition rate via the Purcell effect. This enables higher device operating rates and improves coherence in case the lifetime can be reduced below the dephasing times.

In the following, we develop these concepts by discussing how the photon generation rate can be enhanced and the photon coherence improved by modifying the local density of states. For all discussed solid-state quantum emitters, this can be achieved through advanced nanofabrication techniques, while choosing the best geometry in view of the emitter limitations and the planned device applications.

### 4.1 Emission enhancement via the Purcell effect

Several effects observed with solid-state quantum emitters can impede their performance as single-photon sources: First, they can exhibit nonradiative relaxation channels, which can significantly reduce the efficiency of photon emission. Second, transitions to unwanted levels or via phonon-sidebands (as quantified by the Debye–Waller factor) can lead to a spectrally broadened emission. Finally, the emission frequency may vary over time because of the coupling of the emitter to a fluctuating environment. The impact of the mentioned effects can be reduced when the emitters are integrated into optical resonators [175], as explained in Box 3.

When using resonators with small mode volume and high quality factor, the Purcell effect leads to a lifetime reduction. This has several key advantages: First, it increases the Fourier-limited linewidth; once it becomes broader than the homogeneous broadening and spectral diffusion, this enables the generation of indistinguishable single photons. Second, resonators with a high-quality factor will only enhance the emission to a single resonant level via a zero-phonon transition. This can suppress the relative contribution of the phonon sidebands and unwanted transitions. Third, a reduction of the radiative lifetime can increase the efficiency of emitters with nonradiative decay pathways. Finally, the decay is channeled into a single field mode, which is a key requirement for efficient single-photon sources.

Many different geometries can be used to implement suited resonators for single-photon sources. Some of these resonators feature out-of-plane emission, such as micropillars, circular Bragg gratings (CBGs, also called “bulls-eye” resonators), and Fabry–Perot resonators. Their vertical emission can enable efficient free-space photon extraction or direct coupling to optical fibers. In contrast, in-plane emission is typically obtained with photonic crystal cavities or waveguide-integrated cavities. This enables on-chip light routing and efficient coupling to optical fibers via grating or edge couplers.

**Figure 5:**
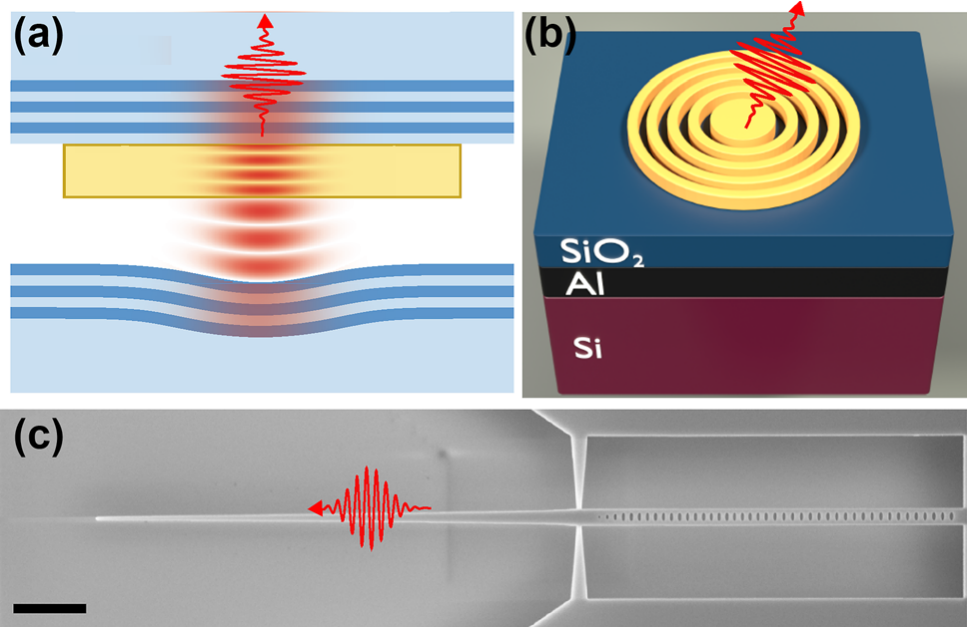
Different types of photonic resonators used with single-photon sources at telecommunications wavelength. (a) Fabry–Perot resonator. A standing-wave cavity mode (red) is formed between two Bragg mirrors of alternating high (dark blue) and low (light blue) refractive index. Photon emitters are integrated into a crystalline slab with a thickness of a few micrometers (yellow). A small mode waist of only a few μm and a large mirror reflectivity, up to 99.999 %, allow for Purcell factors of several hundred. To first order, this is independent of the crystal thickness, such that the distance from the interface can be chosen large enough to preserve the coherence of the emitters. Adapted from [23]. (b) Bulls-eye, or circular Bragg grating cavity. A set of concentric rings is used to confine the light in the plane of a photonic thin film and to shape the mode of the emitted light. With a metal mirror at the bottom, a highly efficient collection can be achieved. The Purcell enhancement in this approach is ≲ 10, making it ideally suited for fast photon emitters such as quantum dots. (c) Nanophotonic resonator. A photonic thin film is patterned to form a waveguide with holes that generate a photonic band gap. Thus, the light can be confined below a single cubic wavelength while keeping quality factors in excess of 10^5^. The resulting lifetime reduction, up to 1000-fold, is required to use otherwise slow emitters such as T-centers or erbium dopants. Adapted from [196].

A summary of the most prominent approaches for efficiently coupling single emitters to an optical field mode is shown in Figure 5.

Box 3: The Purcell effect.

**Figure:**
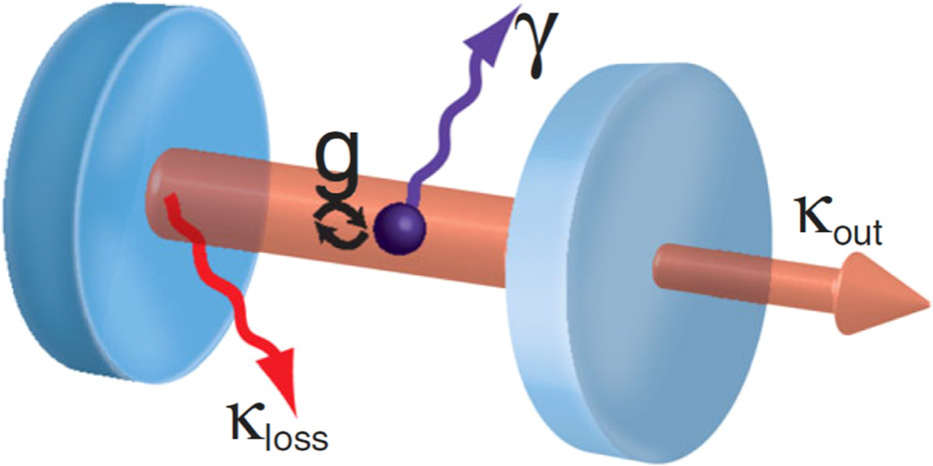

When a single emitter is placed into an optical resonator, its coupling to a single field mode at rate *g* can be larger than the dephasing and polarization decay to other modes at rate *γ*. If the outcoupling rate of the field mode, *κ*_out_, exceeds the absorption and scattering loss of the resonator, *κ*_loss_, the emitted light is channeled into a single propagating field mode, enabling an efficient single-photon source. In addition, the emission rate is increased by the Purcell factor

(4) $$F_{\text{P}}=\frac{g^{2}}{2\kappa \gamma }=\frac{3Q}{4\pi^{2}V}{\left(\frac{\lambda }{n}\right)}^{3}.$$

Here, *Q* is the resonator quality factor, *V* is the mode volume, *λ* is the emission wavelength, and *n* is the refractive index of the resonator. In summary, the Purcell effect thus serves three key purposes:

– increasing the photon emission rate,
– improving the coherence of the emitted photons,
– ensuring that the emission is directed into a single mode.

As can be seen from Eq. (4), achieving a high Purcell factor requires resonators with a high quality factor *Q* and small mode volume *V*. In addition, one needs to

– orient the dipole moment of the emitter parallel to the electric field of the resonant mode,
– ensure that the transition frequency matches the cavity mode frequency,
– place the emitter precisely at the maximum of the field mode.

Figure reprinted from [175].

#### 4.1.1 Optical resonators for erbium dopants

For slow emitters, such as T-centers in silicon and erbium dopants in any host material, achieving a strong Purcell enhancement is a crucial requirement to achieve practical rates. In particular, the ms-long radiative lifetime of the telecom optical transitions of erbium dopants makes it challenging to use them as a coherent quantum light source in spite of their exceptional coherence properties. Even the shortest radiative bulk lifetimes, 142 μs observed recently in silicon [189], exceed those of other emitters discussed in this review by orders of magnitude. Thus, with plain bulk crystals, the count rates are simply too low for experiments and applications. This challenge can only be overcome by integrating the emitters into optical resonators with very strong Purcell enhancement [175]. Currently, ∼ 1,000-fold enhancement is achieved in nanophotonic devices [204] and ∼ 100-fold in Fabry–Perot resonators [23]. This not only enhances the rates but also facilitates the generation of single photons with lifetime-limited optical coherence [23], [200], a key enabler for quantum networking experiments.

#### 4.1.2 Nanophotonic integration of color centers

The nanophotonic integration of color centers in SiC and Si has been a focus of interest in recent years. In SiC, a recent key step was the development of thin film technology [162], which has enabled the fabrication of resonators with small mode volumes and high quality factors. While visible emitters have been integrated into such structures [205], so far experiments with telecom photons are missing. In contrast, this has been achieved with color centers in silicon, where compared to all other materials, nanofabrication is much more advanced because of its prominence in the semiconductor industry. Remarkably, even foundry-based fabrication of low-loss waveguides [203] and resonators with very high Purcell factors [206] is commercially available. Still, the integration of color centers into such structures has only been shown recently.

First experiments have studied single T-centers and G-centers in waveguides [207], [208], [209], [210]. With ensembles of emitters in ring- [211] and CBG [212] resonators, a slight Purcell enhancement of zero-phonon-line emission has been reported, followed recently by the deterministic fabrication of a single W-center in a CBG cavity with the Purcell factor of 7.2 [170]. In nanophotonic “puck” structures, even transitions between the spin levels of individual T-centers were resolved [168]. Finally, recent experiments have studied the interference of photons emitted consecutively from a single emitter in a waveguide [210] or from two emitters in remote cavities [176]. In both experiments, the first signs of indistinguishable photon generation were achieved when the emission was filtered in the time domain such that only small detection-time differences were included in the analysis. To avoid the resulting reduction of efficiency, color centers with better frequency stability or resonators with stronger Purcell enhancement would be required. This is an active field of research, and several groups have recently reported significant lifetime reductions for G- [213], [214] and T-centers [176], [215], [216] in photonic crystal resonators.

#### 4.1.3 Quantum dots

##### 4.1.3.1 Out-of-plane emission designs

One of the main challenges for quantum light sources is achieving a high photon extraction efficiency *η*, defined as the fraction of emitted photons collected by the first lens of the optical system. A typical level for high-refractive index host materials is *η* < 1 % due to the total internal reflection at the air–semiconductor interface. To address this, broadband enhancement of photon extraction could be achieved by engineering the far-field directionality without embedding the emitters into a cavity, for instance, by incorporating a bottom mirror.

The GaAs material system enables a straightforward way to generate distributed Bragg reflectors (DBR) based on sequential deposition of AlGaAs and GaAs layers with a thickness of a quarter wavelength. This approach is compatible with a metamorphic buffer placed in the cavity on top of the DBR [55], which, however, comes at the price of a higher probability of having a dislocation in the QD vicinity. Such a defect in crystal lattice introduces dangling bonds, which act as nonradiative recombination centers. For the InP material system, the DBR has to include more mirror layers than GaAs to obtain a similar reflectivity because of the lower contrast between the refractive indices. A thicker DBR based on quaternary compounds, such as InAlGaAs, requires a laborious, time-consuming growth calibration [54] and significantly increases the device size. Due to these reasons, just a few reports to date feature QDs integrated with an InP-based DBR [39], [64], [100], [217]. An alternative approach is shaping the photonic environment by an optical horn structure [110] or a metallic mirror placed beneath the semiconductor membrane containing QDs realized by wafer bonding. The latter hybridization approach relies on the flip-chip process where the membrane with QDs is placed atop the metallic mirror and a dielectric spacer, all bonded to a carrier wafer (typically silicon). The hybrid design offers an easy and compact solution that circumvents the complexity of DBRs and allows for a comparable total photon extraction efficiency of > 10 % from a single QD [218]. The spectral mismatch problem between the emitter and cavity resonance is eliminated in this approach, in contrast to micropillars or other high-*Q* cavities.

High photon extraction efficiency and Purcell factors for QDs can be achieved by placing them in CBGs. These nanocavities can be etched in the substrate [219], [220], membranized [45], [221], or hybridized [34], [40], [102], [222], [223], [224], [225], [226]. Assuming a typically calculated Purcell enhancement of *F*_P_ = 18 [102], [226], [227], it is possible to collect *β* = (*F*_P_ − 1)/*F*_P_ = 94.4 % of the emitted photons, and to shorten the radiative decay to levels comparable with the exciton coherence time, which is indispensable for many applications in quantum information processing.

##### 4.1.3.2 Integration with photonic circuits

On-chip integration marks a significant step toward a large-scale scalable QIP platform. To realize this, quantum light sources based on epitaxial QDs can be integrated into Si, SiN, or even LiNbO_3_ [228] photonic platforms, which benefit from mature fabrication processes for low-loss optical components. Heterogeneous integration can be achieved through methods like direct wafer bonding [229] or pick-and-place techniques [230], including micro-transfer printing [231], [232]. See also the recent review [233]. The photonic crystal nanobeam cavity is commonly transferred, as it offers high waveguide coupling and the Purcell enhancement of the emitter emission rate [231]. Another explored direction is hybrid integration, where telecom QDs are interfaced with the low-loss SiN platform by photonic wire bonding [234].

While monolithic integration via direct epitaxial growth of III–V compounds on Si presents an intriguing solution [235], [236], [237], [238], this approach has not matured yet and is not competitive with existing technologies.

### 4.2 Deterministic fabrication of devices with telecom quantum emitters

The above-mentioned techniques have enabled the efficient generation of photons at telecommunication wavelengths in many experiments, as summarized in Table 2. The following section will discuss how these devices can be further improved and fabricated deterministically.

**Table 2:** Survey of the state-of-the art achievements with the telecom photon emitters. QD material is InAs, and the growth mode is Stranski–Krastanov unless specified as NW (nanowire), DE (droplet epitaxy), or marked with * (local droplet etching). Designation used: *λ* – wavelength, *η* – photon extraction efficiency (or fiber-coupled efficiency, if marked with FC), *F*_P_ – Purcell factor, *T*_1,opt_ – optical lifetime, TPI – two-photon interference, Ph. C. – photonic crystal, SPH – single photon horn, CBG – circular Bragg grating, DBR – distributed Bragg reflector, SIL – solid immersion lens, NB – nanobeam resonator, F–P – Fabry–Perot, PS – postselected, 2E – TPI from different emitters, nonres. – nonresonant, RF – resonance fluorescence, TPE – two-photon excitation, SUPER – Swing-Up of Quantum Emitter Population [239].

Matrix	*λ* [μm]	Structure	*η* [%]	*F*_P_	*T*_1,opt_	*g* ^(2)^(0)	TPI visibility *V*	Excitation	Ref.
Quantum dots									
InP	1.56	Ph. C.		5	0.2 ns	(0.10 ± 0.02)		Nonres.	[240]
InP	1.58	SPH			1.21 ns	(4.4 ± 0.2) × 10^−4^		*p*-shell	[63]
GaAs, MB	1.55	Planar			1.7 ns	(0.072 ± 0.104)	PS: (89 ± 11) %	RF & TPE	[241]
GaAs	1.30	CBG	23	4	0.3 ns	0.01		*p*-shell	[242]
InP	1.56	DBR	13.3		1.3 ns	0 ± 0.083		Nonres.	[217]
InP	1.54	Mesa	10		1.7 ns	0 ± 0.038		Nonres.	[218]
InP	1.53	Mesa			1.21 ns	(5 ± 4) × 10^−3^	(35 ± 3) %	TPE	[103]
GaAs	1.32	CBG	11.2	4.2	0.3 ns	(0.087 ± 0.003)		Nonres.	[225]
GaAs	1.33	CBG		5.5	0.29 ns	(0.193 ± 0.022)		Nonres.	[226]
GaAs, MB	1.56	CBG	17	4	0.52 ns	(5.2 ± 0.1) × 10^−3^	(8.1 ± 3.4) %	*p*-shell	[220]
InAsP/InP	1.31	NW	27.6		2.1 ns	0.021		Nonres.	[91]
GaSb/AlGaSb*	1.47	SIL			0.43 ns	(0.16 ± 0.02)		Nonres.	[62]
InP	1.55	CBG	16.6	5	0.4 ns	(3.2 ± 0.6) × 10^−3^	(19.3 ± 2.6) %	LO-phonon	[102]
GaAs	1.30	CBG		3.9	0.4 ns	(0.055 ± 0.034)	PS: (61.1 ± 3.2) %	Nonres.	[243]
InP	1.53	CBG	24	5.3	0.78 ns	(0.078 ± 0.016)	PS: (33.4 ± 0.1) %	Nonres.	[244]
GaAs, MB	1.55	CBG		4.3	0.46 ns	(0.076 ± 0.001)	(50.4 ± 1.8) %	SUPER	[245]
GaAs, MB	1.55	CBG		4.3	0.46 ns	(0.069 ± 0.001)	(66.4 ± 0.4) %	LA-phonon	[245]
InP	1.55	NB	12.4		1.87 ns	(0.015 ± 0.003)	PS: (84 ± 6) %	Nonres.	[52]
DE, InP	1.52	Ph. C.		5	0.34 ns	(0.164 ± 0.009)		LA-phonon	[246]
InAlGaAs/InP	1.55	CBG		6.7	0.18 ns	(0.057 ± 0.004)		*p*-shell	[68]
InAlGaAs/InP	1.55	CBG		4.7	0.26 ns	(0.0307 ± 0.0004)	(71.9 ± 0.2) %	*p*-shell	[56]
InAsP/InP	1.27	NW	28		2.35 ns	(0.006 ± 0.003)	5.6 %	Nonres.	[144]
Color centers									
G*:Si	1.27	Ph. C.	29	8	6.7 ns	(0.30 ± 0.07)		Nonres.	[213]
G:Si	1.28	Ph. C.			6.1 ns	(0.03 ± 0.07)		Nonres.	[214]
G:Si	1.28	Planar			4.9 ns	∼ 0.1		Nonres.	[247]
G*:Si	1.28	Planar			33.4 ns	∼ 0.3		Nonres.	[247]
T:Si	1.32	NB	13	6.9	136.4 ns	(0.024 ± 0.018)		RF	[215]
T:Si	1.32	NB		14.6	64.5 ns	(7.6 ± 0.1) × 10^−3^	20 %, 2E	RF	[176]
T:Si	1.33	NB	23, FC	5	168.7 ns			Nonres.	[216]
W:Si	1.22	CBG		7.2	7 ns	0.06		Nonres.	[170]
Erbium dopants									
Er:YSO	1.54	F–P	63, FC	110	104 μs	(0.13 ± 0.02)		RF	[23], [24]
Er:CaWO_4_	1.53	NB	9.4, FC	850	7.4 μs	(0.018 ± 0.003)	(80 ± 4) %	RF	[200]
Er:LiNbO_3_	1.53	NB	5, FC	177	12.5 μs	(0.19 ± 0.02)		RF	[201], [202]
Er:Si	1.54	NB	20, FC	177	0.8 μs	(0.017 ± 0.012)		RF	[196], [197]
Quantum dots in 2D systems									
MoTe_2_	1.54	Exfoliated			1.13 μs	(0.155 ± 0.009)		Nonres.	[92]

So far, most demonstrations of quantum light sources based on QDs, color centers, and erbium dopants employed randomly distributed quantum emitters. This severely reduces the processing yield and technology scalability, as well as the efficiency of light–matter interactions [13], [14], as an effective optical interface between a quantum emitter and a photonic device requires high precision of the quantum emitter alignment to maximize coupling to specific spatial photonic cavity modes (cf. Box 3). Additionally, maximization of the photon coherence time of the quantum emitter requires separation of the emitter from the etched surfaces of the device to reduce the coupling to fluctuating charges at the interface [175], [248].

A solution to this challenge is a deterministic fabrication that can follow two paths: creating the emitter at a precisely defined location or shaping the photonic environment around a quantum emitter preselected from their random distribution. For a broader overview of the topic, we refer readers to the comprehensive review by Rodt et al. [249]. Here, we will focus specifically on the telecom wavelength range.

#### 4.2.1 Position-controlled fabrication

Ideally, quantum emitters should be formed at precise positions to facilitate seamless integration into optical cavities without requiring additional localization steps. For QDs, progress in site-selective QD epitaxy was discussed in Section 3.1.2.4. For color centers and erbium dopants, two approaches exist, as briefly discussed in Section 3.3.2: First, one can locally form and erase color centers in silicon by laser irradiation [171], [172], [208]. This technique will likely be limited by the diffraction limit and thermal conduction to volumes exceeding that of nanophotonic cavity modes. In contrast, local or masked implantation can be used to introduce the emitters at well-defined positions in the host crystal with subwavelength accuracy [169], [170].

**Figure 6:**
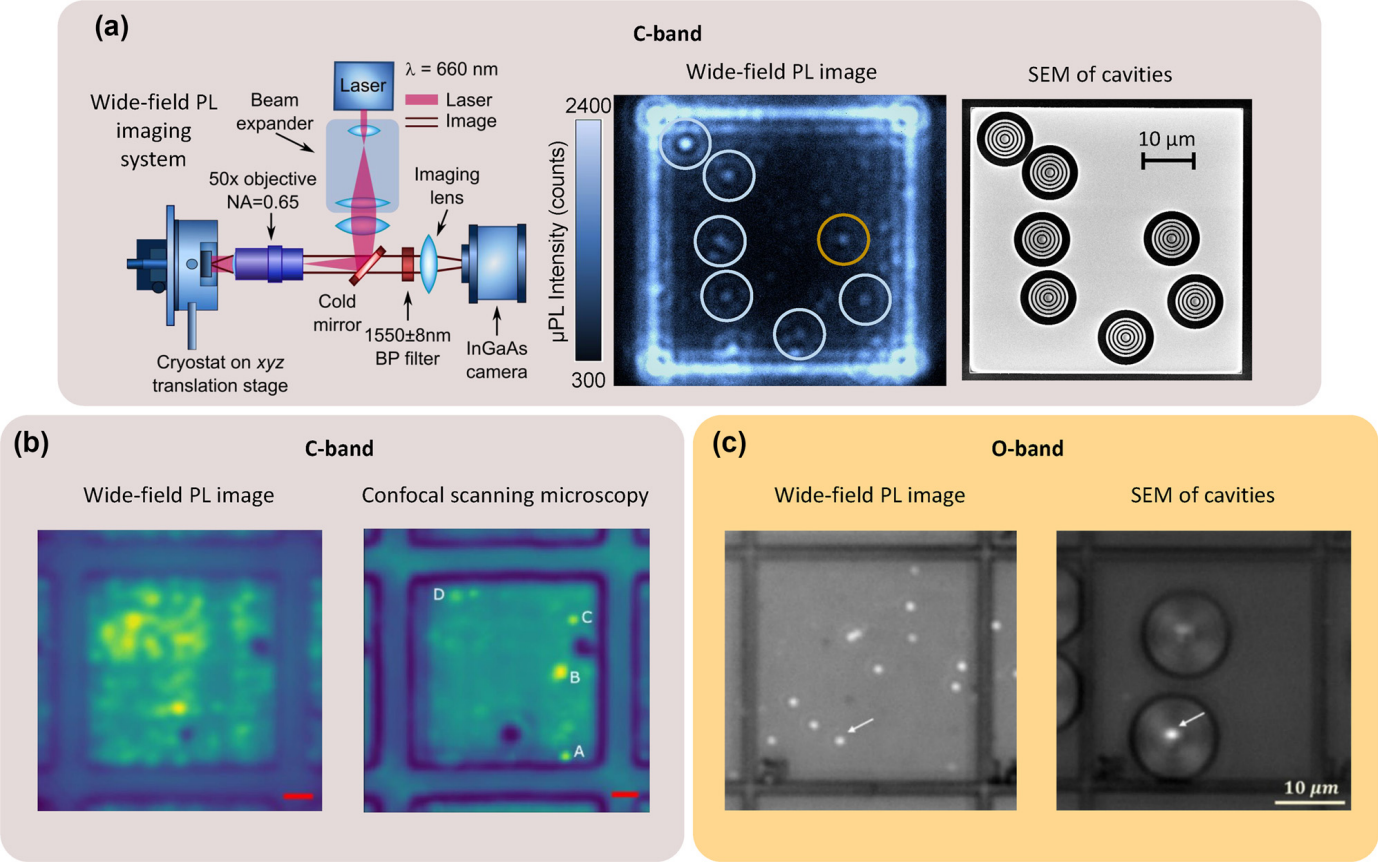
Colocalized fabrication of CBG cavities at the local implantation sites (color center creation sites). (a) The color W-centers are created in silicon-on-insulator (SOI) at well-defined positions by Si^+^ ion implantation through nanoholes in a PMMA mask (top), followed by thermal annealing (center), and fabrication of CBG cavities centered on the nominal coordinates of the nanoholes. (b) PL map of the unpatterned SOI wafer, taken at 10 K. Inset: PL map of a single W-center in an unpatterned SOI wafer. The arrow serves to indicate the idea and does not show the location of an emitter showcased in the inset. Figure adapted from Lefaucher et al. under a CC-BY license [170].

While the first experiments used bulk layers [169], the transfer to nanophotonic resonators has been demonstrated recently [170], achieving the localization of the emitter precisely at the maximum of optical resonators. Figure 6 summarizes the results of colocalized fabrication of CBG cavities at the local implantation sites. However, the number of photon emitters generated in this process is expected to follow a Poissonian distribution. This impedes having *exactly* one emitter in *each* device, and the yield defined in these terms was estimated to ∼40 % [170]. However, if the spectral diffusion linewidth of the emitters is much smaller than the inhomogeneous distribution of the emission frequencies, as often observed with color centers and erbium dopants, single emitters can still be controlled via spectrally selective addressing, as described in Section 3.3.3.

#### 4.2.2 Localization approaches

In the localization approach, optical resonators are fabricated at selected positions of precharacterized emitters. The process thus combines wavelength selectivity during the preselection process with brightness enhancement through cavity integration, offering the fabrication of many identical devices. Localization approaches can be categorized into two main methods: scanning *in situ* and imaging. Scanning involves sequentially scanning the area using a focused electron or laser beam to identify the emitter positions, followed by cavity fabrication one by one, while imaging captures the spatial distribution of emitters in a single step, providing a broader overview. See also the review in [251].

The scanning methods have been used extensively for QD emitters operating at shorter wavelengths. The reported realizations include

1. cathodoluminescence spectroscopy followed by EBL [252], [253] that has been applied to fabricate microlenses [254], microobjectives [255], mesas [252], [256], [257], nanoantennas [258], and CBGs [219],
2. low-temperature laser photolithography [259] with demonstrations including micropillars [28], [260], [261], [262], [263], [264], CBGs [222], and solid immersion lenses [265], and
3. topography scanning using AFM [266] or SEM [267], [268].

However, so far, none of the above-mentioned approaches has been implemented with telecom emitters. Also, PL imaging has so far been used predominantly for the NIR spectral range [34], [45], [223], [269], [270], including the 2nd telecom window [225]. However, recently this approach has been demonstrated in the 3rd window [102], also in the confocal configuration [250]. In this approach, the emitters are localized and selected based on their emission wavelength using a narrow band-pass filter. The spectral distribution of the localized dots is controlled by the selection of the filter bandwidth in order to match the designed cavity. The selection of spectrally suitable QDs opens the way for fabricating large volumes of photonic devices while maintaining consistent quality. The advantage of PL imaging is the rapid data collection compared to scanning techniques and the separation of QD detection and device fabrication steps so that thousands of devices can be fabricated in a single process run. This provides a high-fidelity route to the fabrication of an ensemble of bright quantum light sources with desired properties on a single semiconductor wafer.

**Figure 7:**
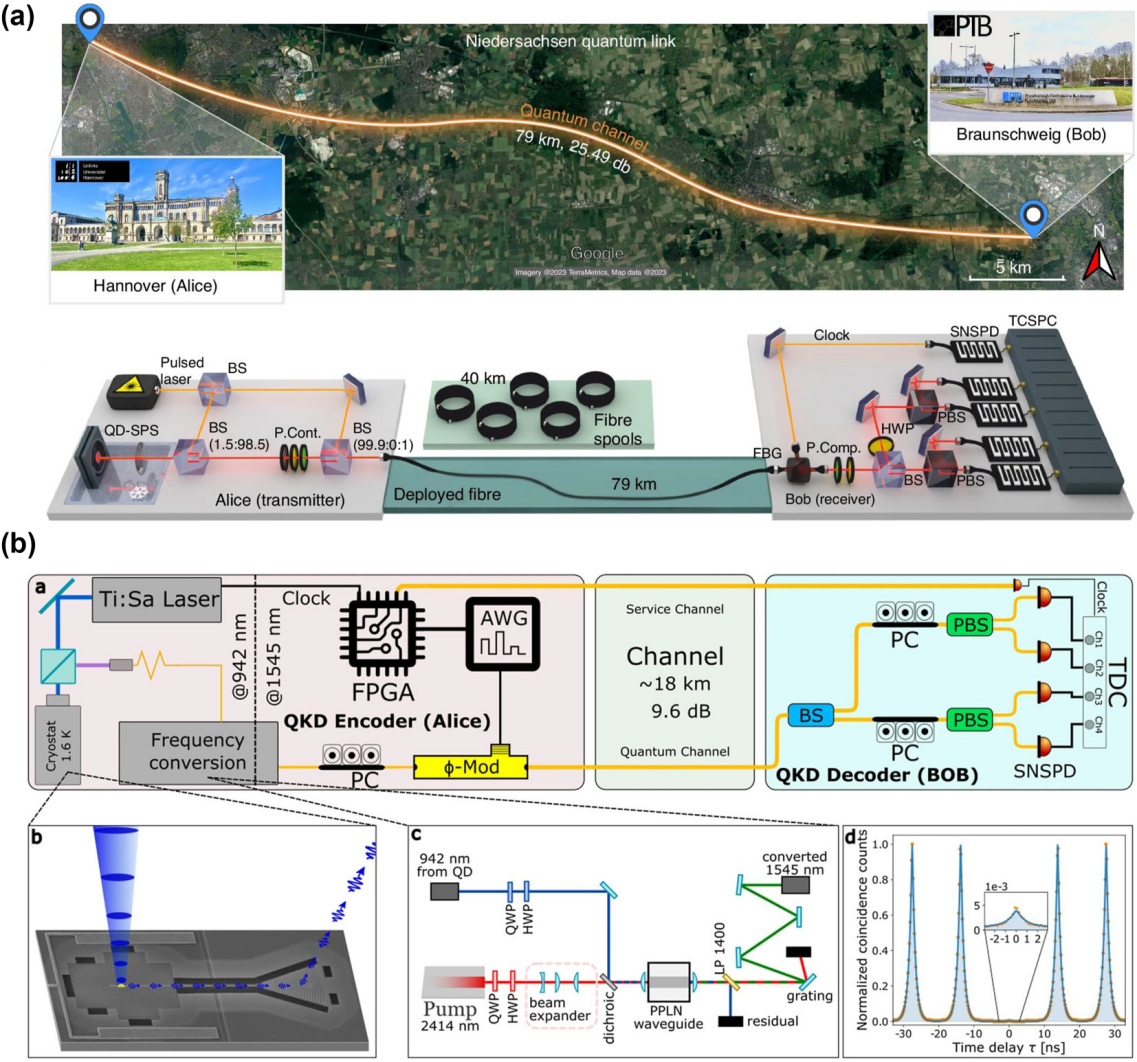
Photoluminescence imaging of quantum emitters at telecom bands. Quantum dots are grown at random positions and are localized optically for the subsequent deterministic fabrication of cavities. (a) PL imaging in C-band using the wide-field imaging system (left), and CBG cavities are deterministically fabricated at the localized QD positions (right). (b) Comparison of the imaging quality for the wide-field PL imaging (left) and confocal laser scanning microscopy (right) in C-band. (c) Wide-field imaging and deterministic fabrication of cavities in the telecom O-band. Panels (a) and (b) are adapted from Refs. [102], [250], respectively, via a Creative Commons Attribution 4.0 International License. Panel (c) is adapted from Ref. [225] with permission of Chinese Laser Press.

A major obstacle to the use of PL imaging at telecom wavelength is the lack of high-quality InGaAs detectors operating at telecom that would have comparable properties to their Si-based counterparts operating in the short-wavelength UV to NIR spectral range. The main limiting factor is the dark current, which is a few orders of magnitude higher for the InGaAs. Despite that, as it has been shown in Refs. [225] and in [102], the imaging process in the telecom spectral range with commercially available InGaAs-based cameras is possible, giving satisfactory results with spatial positioning accuracy of a few tens of nanometers. Figure 7 shows the result of PL imaging using wide-field illumination in the C-band [102] and O-band [250], and the comparison of the PL signal collected in this method with confocal laser scanning microscopy in the C-band [225].

### 4.3 Comparison of the different emitters

The previous sections described the key properties of the different quantum emitters discussed in this review. They are at different stages of their development, face different challenges, and have different levels of application readiness. To allow for a direct comparison, Table 3 summarizes the key properties and provides an overview of the current state of the literature on telecom-wavelength single-photon sources.

**Table 3:** Comparison of the characteristics of the emitters described in this review.

Characteristics	Quantum dots	Color centers	Erbium dopants
Fabrication method	Epitaxial semiconductor growth	Implantation or integration during growth	Implantation, in-diffusion, or integration during growth
Host material	III-V semiconductor: GaAs, InP, and their compounds	Si and SiC	Si [196], Y_2_SiO_5_ [23], [181], CaWO_4_ [200], LiNbO_3_ [201], [202], and others
Theoretical description	Well-described electronic structure: 8-band k⋅p [271], [272], [273] or atomistic tight-binding theory [274];	Density-functional-theory [151], different levels of maturity for different centers;	Crystal field Hamiltonian analysis [180], different levels of maturity for different hosts;
Accessible telecom wavelength range	1.3–1.6 μm	1.21–1.33 μm	1.53–1.54 μm
Typical bulk lifetimes	∼1–3 ns [52], [63], [91], [217], [218]	≳10 ns to 1 µs [160], [161], [213]	From ∼0.14 ms [196] to ∼11.4 ms [23]
Achieved Purcell factor	∼ 6.7 [68]	∼ 29 [213]	∼ 850 [200]
TPI visibility	72 % [56]	≲20 % [176]	(80 ± 4) % [200]
Optical transition coherence	380 ps [56]	a few ns [161], [176]	110 μs [23] (lifetime-limited in resonator)
Demonstrated electronic spin coherence	240 ps [50]	2.1 ms [160] (T-center in isotopically purified Si)	23 ms [192] (Er in CaWO_4_)
Nuclear spin coherence	–	220 ms (single spin) [176]	>1 s [193] (ensemble)

Based on these differences, it becomes clear that the systems have distinct advantages and disadvantages, also depending on the targeted application. The mature fabrication of quantum dots and their high brightness have made them prevalent for applications in the field of quantum communication, which will be discussed in the next sections. However, the absence of memories with long coherence times currently prevents long-distance spin–spin entanglement as required, e.g., for quantum repeaters. In addition, the integration of QDs into photonic circuits remains challenging, as they are typically grown in III–V semiconductor systems and thus require heterogeneous integration with passive low-loss platforms such as silicon or silicon nitride.

In contrast, silicon-based emitters, such as color centers and erbium dopants, can be directly generated or implanted into the crystal of which the photonic integrated circuit is fabricated. In addition, erbium dopants benefit from a unique electronic structure that shields them from interactions with the surrounding lattice, minimizing spin and optical dephasing. However, dopant integration and deterministic fabrication of color centers by ion implantation remain an active area of research. In addition, many of these emitters still require fundamental studies to refine their formation, their theoretical description, and their electronic structure.

Table 4 provides a comparative overview of the current state of the systems discussed in this review, summarizing their respective advantages and disadvantages.

**Table 4:** Comparison of the advantages and disadvantages for each of the systems discussed in this review.

System	Advantages	Disadvantages
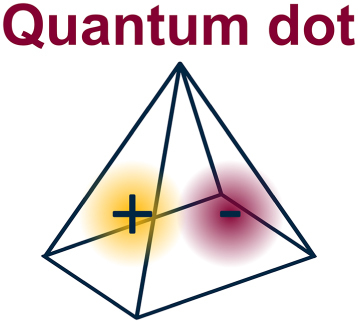	– Short radiative lifetimes → high photon flux	– Site-controlled growth challenging
	– Demonstrated high photon indistinguishability	– Integration with low-loss photonic circuits requires heterogeneous approaches
	– Mature fabrication technology → complex photonic and electronic device functionalities	– Short spin coherence time
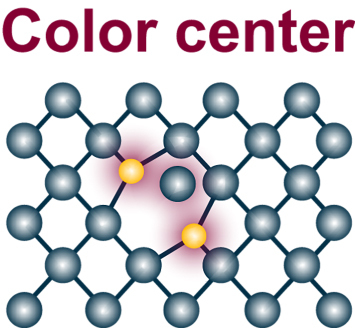	– Fabricated directly in the host lattice	– So far, moderate optical coherence in the telecommunications bands
	– Long spin coherence time	– Challenging to generate on a single-emitter level
	– Moderate or low inhomogeneous broadening	– Spectral diffusion in nanostructures exceeds lifetime-limited linewidth
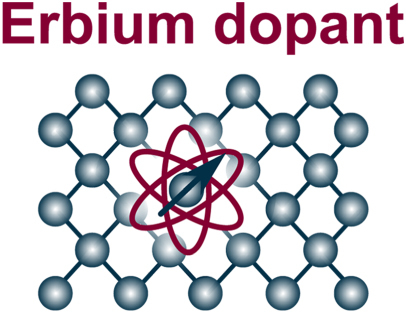	– Directly integrated into host lattice	– Long radiative lifetime
	– 4f screening enables lifetime-limited optical coherence in resonators	– Detection of single emitters requires strong Purcell enhancement
	– Long spin coherence time and low inhomogeneous broadening	– Spectral diffusion so far exceeds the lifetime-limited linewidth
	– Narrow linewidth enables spectral multiplexing	

## 5 Applications of solid-state quantum light sources

The solid-state quantum emitter platforms introduced in the previous sections are promising candidates for various applications in quantum information technology. This section presents an overview of applications demonstrated already using telecom-wavelength quantum emitters and discusses prospects for applications that might become possible in the near or far future. Applications to be discussed in the following concern quantum key distribution, single-photon and entanglement distribution, as well as quantum teleportation. Each section will begin with a brief introduction explaining the background and relevance of the respective field.

### 5.1 Quantum key distribution

Quantum key distribution (QKD) is the most developed and explored application in quantum information with the highest short-term application potential. It enables the establishment of a secret, random bit-string shared between two authenticated parties to be used for data encryption. In contrast to classical cryptography, QKD provides security protected by the laws of quantum physics rather than computational complexity. Using the one-time-pad scheme for data encryption, even information-theoretical security is possible [275], [276].

In 1984, Charles H. Bennett and Gilles Brassard proposed the first QKD protocol [277], today known as BB84, which uses the quantum mechanical properties of single photons to establish a secret key between two authenticated parties. In BB84-QKD, classical information is encoded in the state of flying qubits, e.g., using different polarizations of single photons, in a so-called prepare-and-measure type configuration. The sending party, Alice, randomly prepares qubits in four different states (of two conjugate bases) and sends them to the receiving party (Bob) via a quantum channel. Bob then detects the states in a randomly chosen basis. In this setting, attempts of eavesdropping by a spy lead to increased errors in the transmitted bit string and can thus be detected by comparing a subset of the results. Implementing the BB84 protocol in five basic steps, i.e., photon transmission, key sifting, parameter estimation, and classical postprocessing (error correction and privacy amplification), a secret key can be distilled with the secure key rate and the quantum bit error ratio or rate (QBER) (unit % or s^−1^) as important figures of merit. As the steps mentioned above require an authenticated classical channel between the communicating parties to begin with, QKD is also referred to as a “secret growing scheme.”

While the first single-photon-based implementation of QKD by Waks et al. in 2002 employed a QD source emitting at a wavelength of 880 nm [278], the field soon also tackled implementations in the second and third telecom window (O- and C-band) as discussed in the following.

QKD with single photons at telecom wavelengths was first demonstrated in 2009 using a QD-micropillar cavity emitting at 1,300 nm under above-band excitation [279] – work that built on pioneering earlier work on the QD source itself [280]. In their implementation, the authors choose phase encoding using path-length matched Mach–Zehnder Interferometers at Alice and Bob, to avoid polarization-state distortions in the 35 km long standard SMF-28 optical fiber serving as a quantum channel. Operating this QKD system at a clock rate of 1 MHz, the authors achieved a calculated maximum secure key rate of about 160 bit/s in the asymptotic limit with a measured QBER of 5.9 % and further observed a positive key rate at a distance of 35 km. Moreover, it was shown that the QKD performance achieved with a single-photon source surpassed an analog implementation using an attenuated laser (WCPs without decoy states) in terms of the distance limit.

The first implementation of single-photon QKD in the telecom C-band (1,560 nm), i.e., at the lowest transmission loss possible in optical fibers, was reported only 1 year later [281]. The authors employed a QD integrated into a horn structure (cf. Ref. [110]) and phase-encoding for the protocol implementation. The authors achieved a maximum secure communication distance of 50 km based on the asymptotic GLLP rate equations. Further improving their QKD implementation by employing low-noise single-photon detectors based on superconducting nanowires [282] and a QD source with a lower multiphoton probability (*g* ^(2)^(0) = 0.005), the same group presented an improved version of their QKD implementation in Ref. [283]. Here, the improvements resulted in a maximal communication distance of 120 km – the longest transmission distance achieved in fiber-based single-photon QKD to date.

**Figure 8:**
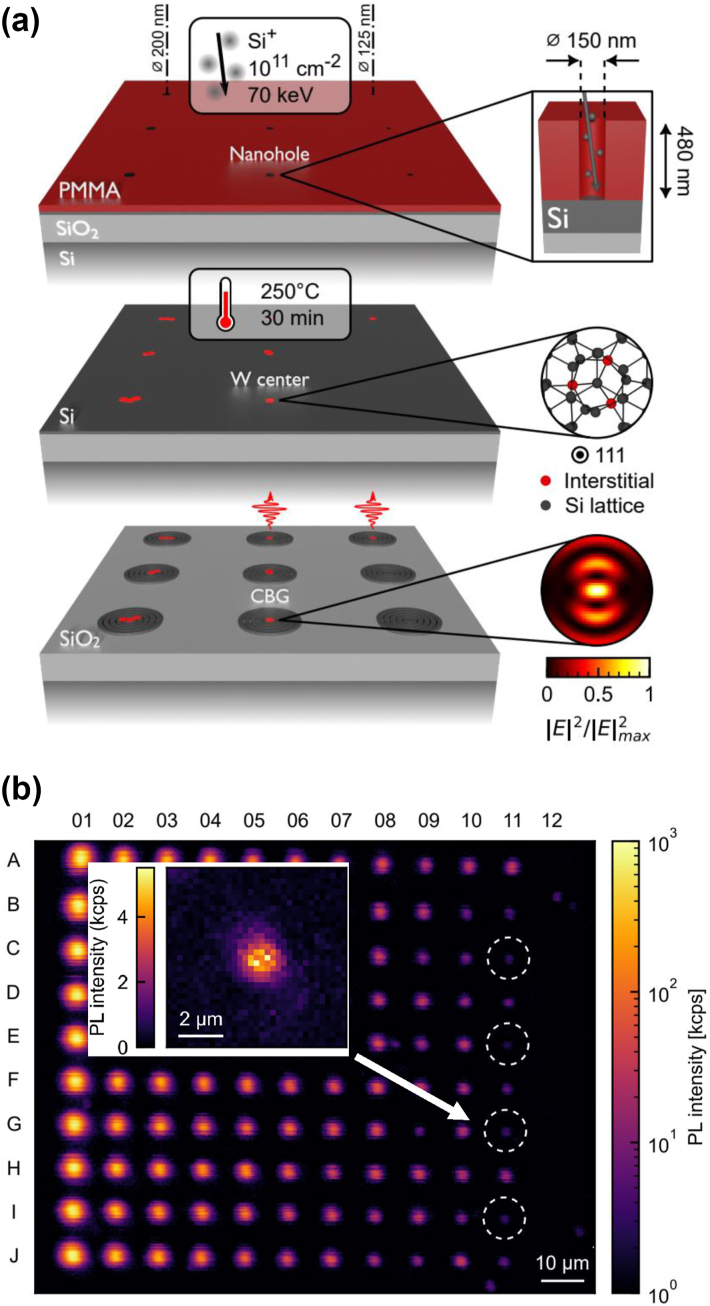
Fiber-optical links for QKD with static and dynamic polarization switching. (a) Test-experiments toward QKD in an intercity fiber-optical link using a QD single photon source emitting at 1,555.9 nm. (b) Experimental layout of a QKD field experiment based on a QD single-photon source quantum frequency converted from 942 nm to 1,545 nm using dynamic, random polarization-state switching on Alice side and a deployed optical fiber link in the Copenhagen metropolitan area serving as quantum channel. Panel (a) is adapted from Ref. [284], and panel (b) from Ref. [285] via a Creative Commons Attribution 4.0 International License.

More recent work reported test experiments toward QKD in an intercity fiber link using a deterministic single-photon source emitting at telecommunication C-band [284]. The 79 km long fiber-optical link with a total attenuation of 25.49 dB is deployed between the German cities of Hannover and Braunschweig (see Figure 8a). Using a QD embedded in a circular Bragg grating structure, the authors emulated the BB84 protocol using static polarization switching and calculated that a secret key fraction of 4.8 × 10^−5^ as well as an asymptotic maximum tolerable loss of 28.11 dB would be possible in a full implementation including dynamic, random polarization switching. Moreover, an average quantum bit error ratio of 0.65 % was observed.

Despite the enormous progress seen in the development of telecom-wavelength SPSs (cf. Section 3), the fabrication of devices offering high performance remains challenging. Therefore, recent work also considers quantum frequency conversion to transfer the emission of high-performance QD-SPSs emitting around 900 nm to C-band wavelengths [49]. Respective sources were first employed for QKD experiments emulating the BB84 protocol using static polarization switching [286]. Following this route, also QKD was demonstrated with dynamic polarization switching and an intracity link consisting of 18 km deployed optical fiber with 9.6 dB channel attenuation (see Figure 8b), which resulted in a secret key rate of 2 kbit/s [285]. This QKD field experiment was located in the Copenhagen metropolitan area and used dynamic, random polarization switching.

**Figure 9:**
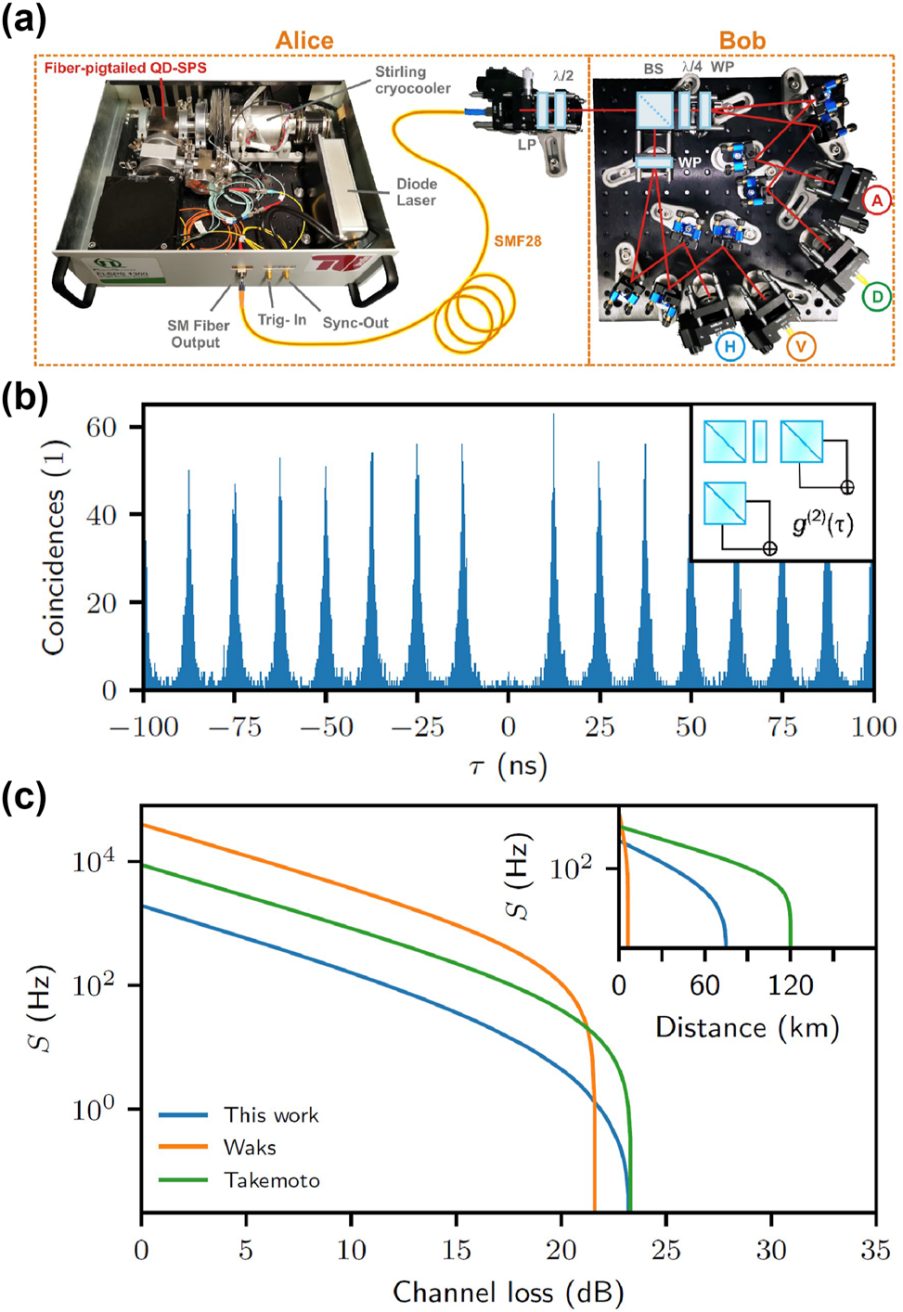
QKD testbed using a benchtop plug&play telecom-wavelength QD SPS providing single-photon pulses via an SMF28 optical fiber for polarization encoding. (a) The 19-inch rack module houses a compact Stirling cryocooler, including the fiber-pigtailed QD device, a pulsed diode laser, and a fiber-based bandpass filter. (b) Photon autocorrelation histogram *g* ^(2)^(*τ*) on the fiber-coupled QD-emission confirming the single-photon nature of the photon stream coupled to the quantum channel. (c) Asymptotic secret key rate *S* versus loss revealing the performance of the plug&play system (blue) compared to early bulky laboratory-scale QKD-implementations reported by Waks et al. (orange, Ref. [278]) and Takemoto et al. (green, Ref. [283]). Adapted with permission from Gao et al., Appl. Phys. Rev. 9, 011412 (2022), Ref. [287]. Copyright 2022, AIP Publishing LLC.

The QKD experiments discussed above relied on full-fledged bulky laboratory infrastructure, preventing field trials in more realistic environments. Combining a compact cryocooler with a fiber-coupled single-photon source emitting at telecom O-band wavelengths, a benchtop single-photon QKD testbed operated with QD-sources was demonstrated [287] (see Figure 9). The plug&play device emits single-photon pulses with an antibunching of *g* ^(2)^(0) = (0.10 ± 0.01) at 1,321 nm based on a directly fiber-pigtailed deterministically fabricated QD mesa structure integrated into a compact Stirling cryocooler in a 19-inch server-rack module. Emulating the BB84 protocol with static polarization switching, the authors observed a raw key rate of up to (4.72 ± 0.13) kHz and a predicted maximal tolerable loss in the asymptotic limit of up to 23.19 dB. Here, a key-rate optimization routine based on two-dimensional temporal filtering was applied to optimize the achievable tolerable loss, as introduced by the authors in prior work [288].

Currently, Stirling-type refrigerators are the most compact solution to operate quantum devices at temperatures down to approximately 27 K, which is sufficient for some applications, including BB84- or E91-type QKD protocols. Applications with more stringent demands for the coherence properties and indistinguishability of the generated flying qubits require 4 K Gifford–McMahon cryocoolers in combination with compact compressors.

While compact cryogenics can be used to enable the field deployment of solid-state single-photon sources as discussed above, ultimately, the room-temperature operation would improve the practicality and substantially reduce the costs. Albeit some solid-state-based quantum emitters can work up to room temperature [11], [12], achieving this in combination with telecom wavelengths is a challenge that was first mastered in 2018. Back then, single-photon emission at O-band wavelengths up to room temperature was observed for point defects in GaN thin films on sapphire [289] and cubic silicon carbide (3C–SiC) [156].

Using the GaN-based single-photon source [289], recently two QKD experiments were reported. The first work describes the implementation of polarization-encoded BB84-QKD in field experiments in deployed optical fibers [290]. Using a 3.5 km (32.5 km) long quantum channel with an attenuation of 4.0 dB (11.2 dB), secure key rates of 585.9 bit/s (50.4 bit/s) are observed at a quantum bit error ratio (QBER) of approximately 5 %. In the second work [291], the same GaN-based source was used to demonstrate both the BB84 protocol and a reference-frame-independent QKD (RFI-QKD) protocol [292]. Both implementations employed time-bin and phase encoding and were realized in a 33 km long fiber-spool (10.44 dB loss) as well as a 30 km long commercial deployed optical fiber (15.52 dB loss). The experiments resulted in a similar performance for the BB84- and the RFI-protocol with a secure key fraction of up to 7.58 × 10^−7^ per pulse, which corresponds to a secure key rate of 60.64 bit/s at the used clock rate of 80 MHz. Interestingly, this result, therefore, also reveals a similar performance of the time-bin and phase coding compared to the polarization-encoding from their first work discussed above. While reporting an impressive performance for room-temperature sources, significant improvements in terms of efficiency and achievable clock rate are required to compete with state-of-the-art cryogenic sources.

Noteworthy, all QKD-implementations using deterministic quantum light sources emitting at telecom wavelengths reported to date used protocols in prepare-and-measure type settings (BB84-type protocols), for which single photons are sufficient as quantum resource. Experiments on entanglement-based QKD-protocols (cf. E91 and BBM92) have been reported for QD-based sources emitting polarization-entangled photon pairs at shorter wavelengths [293], [294], [295]. Given the progress seen in this field, as discussed in the following two subsections, a transfer to entanglement-based QKD protocols can be expected very soon.

### 5.2 Entanglement distribution

Entanglement and its distribution across large distances is one of the most crucial resources for many applications in the context of quantum technologies and quantum communication and networking. The entanglement can thereby be established between two or multiple flying qubits or between stationary and flying qubits. While many experiments in this context have been demonstrated in pioneering work at shorter wavelengths [36], [42], [296], [297], proof-of-concept experiments in the telecom wavelength range are relatively recent.

**Figure 10:**
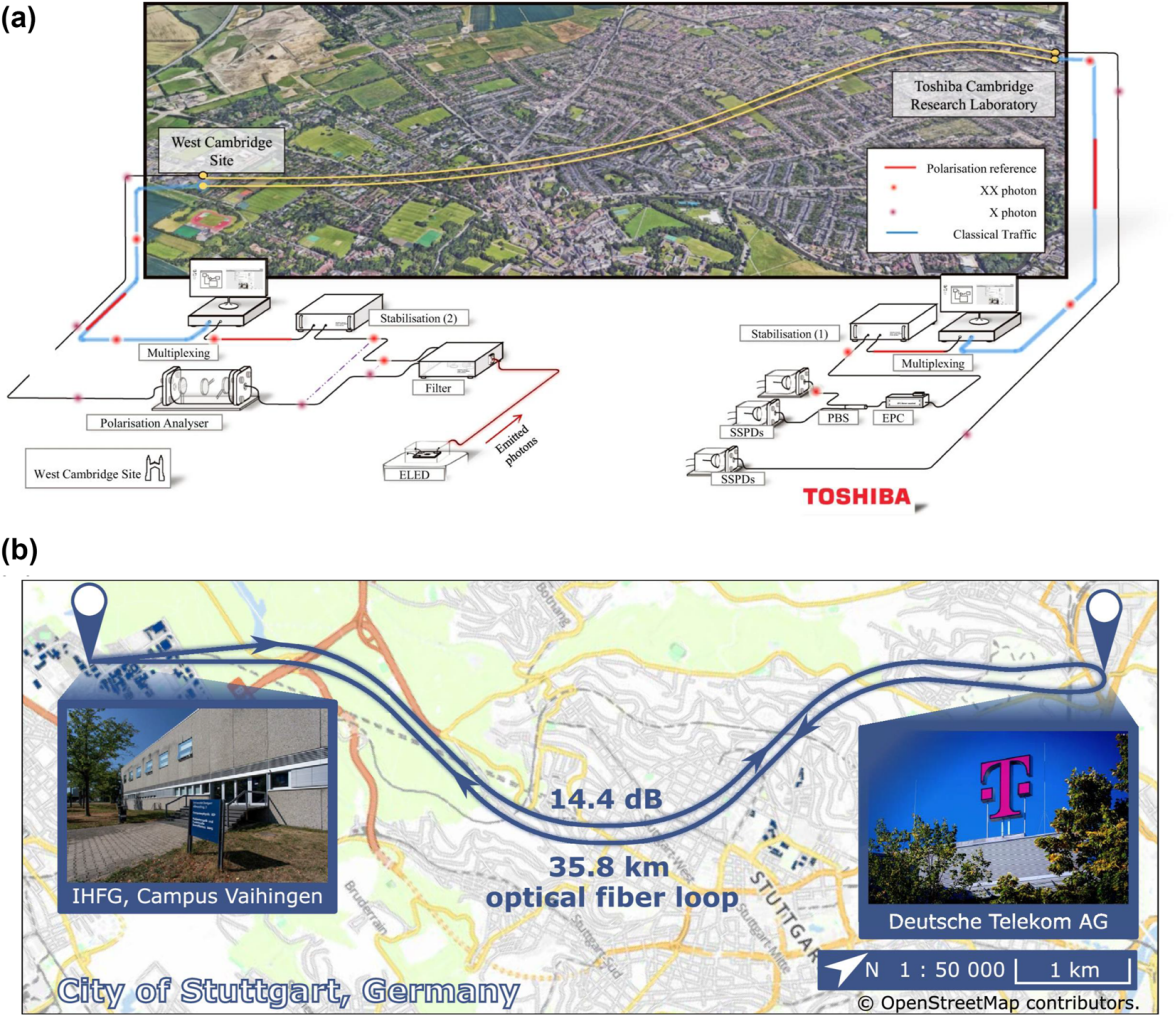
Fiber-optical links for entangled photon transmission. (a) Illustration of a field experiment for entangled photon transmission in a fiber-optical network in Cambridge, England, using a QD-based entangled-light emitting diode emitting around 1,310 nm. Entangled photon pairs are generated by a spectrally tunable entangled-light emitting diode featuring on-chip optical pumping at West Cambridge. The quantum-state measurement at the Cambridge Research Laboratory (CRL), after propagation through 15 km of optical fiber, revealed a high entanglement fidelity. (b) Intracity connection in Stuttgart, Germany, used for entanglement distribution with a QD photon-pair source emitting at 780 nm based on the biexciton–exciton radiative cascade. While one photon was detected immediately, the second one was quantum frequency converted to 1,515 nm before propagating to Stuttgart Feuerbach and back to the research laboratory via a 35.8 km long fiber-optical loop (14.4 dB transmission loss). Panel (a) is adapted from Ref. [300], and panel (b) from Ref. [301] via a Creative Commons Attribution 4.0 International License.

The distribution of entanglement from a telecom quantum light source emitting at 1,310 nm has been demonstrated in 2019 [298] using a QD-device emitting polarization-entangled photon pairs around 1,310 nm – a device used also for experiments on a quantum relay 2 years earlier [299] (see Section 5.3). Shortly after, the same research team also used a fully electrically operated entangled-light emitting diode based on QDs, which featured a wavelength tunability exceeding 25 nm for similar experiments [300] (cf. Figure 10a). Employed in field-trails using a 20 km long fiber-optical intracity link in Cambridge, England, the authors achieved an entanglement fidelity above 94 % and additionally demonstrated spectral multiplexing of single entangled photons with classical data traffic at 1,550 nm.

Recently, entanglement distribution was also realized by quantum frequency converting a QD-source from 780 nm to telecom C-band wavelength [301] (cf. Figure 10b). Here, the authors first confirmed the preservation of polarization entanglement after quantum frequency conversion with Bell state fidelity of (97.2 ± 0.3) % for the biexciton transition. After one photon of the entangled pair had propagated through a 35.8 km long deployed optical fiber, still a high fidelity of (94.5 ± 0.5) % was observed. In addition, the authors demonstrated a second polarization- and entanglement-preserving conversion from 1,515 nm to 780 nm, which showed prospects for the flexible interfacing of quantum light sources with different types of quantum memories.

Another application using a telecom-wavelength photon pair source was presented in 2022 [302], however, without exploiting the entanglement itself. The authors reported a testbed for the distribution of single photons using a QD source emitting in the telecom C-band in a 20 km long optical fiber deployed in the Stockholm metropolitan area. Distributing the photons emitted via the XX-X radiative cascade, the authors demonstrated the distribution of random numbers passing the NIST test suite SP800-22 at a subscriber 8 km outside of the city with a bit rate of 23.4 kbit/s.

Very recently, the first proof-of-concept experiments were reported for spin-photon entanglement using a QD- and an erbium-based quantum light source directly emitting at telecom C-band wavelength. In the case of the first experiment, the authors used a negatively charged exciton in an InAs/InP QD to implement an optically active spin qubit to demonstrate spin-photon entanglement with a fidelity of (80.1 ± 2.9) % [303]. The second experiment used a single Er^3+^ ion in a solid-state crystal, integrated into a silicon nanophotonic circuit, and observed a spin-photon entanglement fidelity of (73 ± 3) % over 15.6 km of optical fiber [304].

Table 5 summarizes applications demonstrated with telecom-photons from deterministic quantum light sources reported to date. Note that in this context, we deliberately decided to also discuss experiments using quantum frequency conversion from QDs emitting at shorter wavelengths (as indicated by arrows), which reflects that the field is currently still in a transition phase toward full functionality at telecom wavelengths.

**Table 5:** Applications of deterministic quantum light sources at telecom wavelengths in QKD, entanglement as well as random number distribution, quantum relays, and quantum teleportation. The figure of merits are the (secure) key rate in (s)bps and the quantum bit error ratio in %, the rate of random number generation, and the teleportation fidelity (abbreviations: Plug&Play (PnP), random number distribution (RND), entanglement distribution (ED), photon (ph.), spin (sp.), quantum frequency conversion (→), polarization (Pol), fiber-coupled (FC), photonic integrated circuit (PIC)).

Application	Emitter/Device	Material	*λ* (nm)	Coding	Fiber channel length	Ref.
QKD	QD/Micropillar	InAs/GaAs	1,300	Phase	Lab-scale	[279]
QKD	QD/Opt. horn	InAs/InP	1,580	Phase	50 km (spool)	[281]
QKD	QD/Opt. horn	InAs/InP	1,500	Phase	120 km (spool)	[283]
QKD test	QD/PnP module	InAs/InP	1,321	Pol	Lab-scale	[287]
QKD test	QD/Micropillar	InAs/GaAs	940 → 1,550	Pol	170 km (spool)	[49]
QKD test	QD/CBG	InAs/GaAs	1,555.9	Pol	79 km (depl.)	[284]
QKD	QD/PhC	InAs/GaAs	942 → 1,545	Pol	18 km (depl.)	[285]
QKD	Point Defect	GaN	1,309.5	Pol	33 km (depl.)	[290]
QKD	Point Defect	GaN	1,305.4	Time/Phase	30 km (depl.)	[291]
RND	QD/-	InAs/InGaAs	1,550	–	20 km (depl.)	[302]
ED (ph.-ph.)	QD/Diode	InAs/InGaAs	1,310	Pol	18.23 km (depl.)	[298]
ED (ph.-ph.)	QD/Diode	InAs/GaAs	1,310	Pol	15 km (depl.)	[300]
ED (ph.-ph.)	QD/Diel. antenna	GaAs/AlGaAs	780 → 1,515	Pol	35.8 km (depl.)	[301]
ED (sp.-ph.)	QD/Planar cavity	InAs/InP	1,533	Pol	Lab-scale	[303]
ED (sp.-ph.)	Er^3+^/PIC	Er^3+^:CaWO_4_	1,532.6	Pol	15.6 km	[304]
Relay	QD/Diode	InAs/InGaAs	1,325	Time	Lab-scale	[299]
Teleportation	QD/Planar cavity	InP/InAs	1,550	Time	Lab-scale	[65]
Teleportation	QD/Diel. antenna	GaAs/AlGaAs	780 → 1,515	Pol	Lab-scale	[305]

### 5.3 Quantum teleportation

Quantum teleportation, enabling the transfer of an unknown quantum state of one particle to another distant particle [306], is an elementary building block for quantum networks and of a particularly intriguing nature, enabling nonlocal quantum computations and the transfer of qubit states into quantum memories [307]. In the case of a photonic implementation of quantum teleportation, the resources required are an entangled photon pair shared between two remote locations associated with Alice and Bob, a projective Bell-state measurement, and the exchange of two bits of classical information. First, Alice projects the state that should be teleported into the Bell basis by performing a Bell-state measurement between her half of the entangled photon pair and the unknown state. The measurement outcome determines the unitary operation that has to be applied in the second step on Bob’s site to the second half of the entangled state, enabling the retrieval of the teleported state. Thus, two bits of classical information are used to communicate the unitary required on Bob’s side.

In the context of quantum communication, teleportation can be used to transfer an encoded state from Alice to Bob with the security of the quantum channel being unconditionally guaranteed [308], even when the entanglement resource is provided by an untrustworthy third party, a functionality referred to as quantum relay.

Building on earlier work at shorter wavelengths [309], the first demonstration of a quantum relay operating in the telecom O-band was reported in 2017 using a sub-Poissonian solid-state quantum light source [299]. The implementation used a continuously excited QD emitting polarization-entangled photon pairs around 1,325 nm and a spectrally tunable attenuated laser encoded in four different polarizations as input states. In this standard four-state protocol, a teleportation fidelity of (87.9 ± 1.1) % (up to 94.5 % with stronger temporal post-selection) was achieved, which exceeds both the classical limit (2/3) and the threshold for error correction required for QKD (80 %).

Three years later, the same research group reported the first QD-based proof-of-concept teleportation experiment operated at telecom C-band wavelengths [65]. Using a QD entangled-photon pair source emitting around 1,550 nm in combination with a time-bin encoded attenuated laser pulse as input state, the authors observed a mean teleportation fidelity of (82 ± 1) %. Noteworthy, these experiments have been realized at GHz clock rates, underlining the prospects of QD sources for high-speed applications.

In 2024, two groups independently reported the first quantum teleportation experiments in which flying qubits generated by two remote deterministic solid-state quantum light sources were used both as entanglement resource and input state for the teleportation [305], [310].

The first experiment reported in Ref. [305] used two remote QD-based sources emitting at slightly different wavelengths around 780 nm, which were coherently excited via pulsed two-photon resonant excitation of the XX–X radiative cascade. Using quantum frequency conversion, the XX-photons from both quantum emitters were converted to 1,515 nm. While one photon served as input for the Bell-state measurement, the other one was the photon to be teleported. The unconverted X-photon of the second quantum emitter was used as a flying target qubit for the teleportation process. Using this setup, the authors observed a postselected teleportation fidelity of up to (72.1 ± 3.3) %, just above the classical limit.

The second experiment reported in Ref. [310] was fully carried out at an operation wavelength of 780 nm using two remote QD sources. In this case, the precise wavelength matching of the sources was achieved using piezo-induced strain tuning of one of the sources.

Both reports mark an important advance for the semiconductor platform as a source of quantum light, fulfilling a key requirement for a scalable quantum network, with entanglement swapping being the next logical step. Demonstrating these types of experiments with multiple remote sources directly emitting at telecom wavelengths will be a major step in the quest for efficient quantum networks and the interfacing of remote quantum computers, as unavoidable additional losses by the conversion process are circumvented.

Looking at Table 5, most applications demonstrated in the field of this review to date have been realized with QD sources. As discussed also in the context of the comparison of the different emitter platforms in Section 4.3, this mainly reflects the high level of maturity in technology readiness of QDs compared to the other platforms. As individual emitter platforms advance over time, color centers and rare-earth dopants will also become increasingly interesting for applications, especially due to their excellent spin and coherence properties, which are particularly relevant for the realization of spin-photon interfaces and quantum memories.

## 6 Current challenges and open questions for telecom single-photon sources

The main challenges in the up-scaling of telecom emitters currently lie in the coherence time, which influences the photon indistinguishability, and in the spatial and spectral distribution of the emitters, which impedes deterministic fabrication and thus hampers the scalability of the systems. This section identifies the main challenges and summarizes current approaches to address them.

### 6.1 Coherence and photon indistinguishability of current devices

The generation of indistinguishable photons from independent quantum light sources is a key challenge for all solid-state single-photon emitters but a prerequisite for many key building blocks for photonic quantum technology, including photonic quantum gates and Boson-sampling devices [311]. The performance of current emitters, summarized in Table 2, still requires significant improvements to enable the up-scaling to many-photon devices. Thus, the following sections will focus on the current approaches to improve photon coherence.

#### 6.1.1 Telecom color centers

Single color centers that emit at a telecommunications wavelength have only been demonstrated very recently. Hence, only a few experimental studies investigated the coherence properties of the emitted photons so far. A first experiment studied subsequently emitted photons from a single G-center in a silicon nanophotonic waveguide [210]. A second recent work investigated the interference of light emitted by spatially separated T-centers [176]. Both experiments observed a high interference contrast only at very short detection time delays (postselection). Thus, the required temporal filtering severely impedes the up-scaling of devices. As the same color centers in bulk crystals can exhibit much narrower optical lines [160], the noise in nanophotonic devices is probably caused by the proximity of interfaces, where trap states change their charge and spin during optical excitation and thus cause instantaneous spectral diffusion. Similar difficulties have been observed earlier with color centers in diamond, and a strong improvement was obtained by using emitters that are – to first order – insensitive to electric field noise [151]. Finding such emitters at telecommunication wavelengths is an open challenge.

#### 6.1.2 Erbium dopants

In spite of the insensitivity of the 4f transitions to perturbations, erbium dopants that are integrated into nanophotonic devices also suffer from the proximity to interfaces. This can be seen by comparing measurements of the spectral diffusion linewidth of single emitters in the same host material: In the proximity of an interface, the observed linewidth of ≳10 MHz [181] is almost hundredfold larger than that found in a 20 μm-thick, bulk-like membrane [23], [24], which was found to be limited by the coupling to a nuclear spin bath rather than by the interface.

Recently, two key steps have been demonstrated to overcome this challenge. First, it has been shown that the entanglement of remote emitters does not require photon indistinguishability as long as the emission frequency is constant over extended periods of time [25]. Second, it has been demonstrated that stable emission frequencies can be achieved in Fabry–Perot resonators [23] and even in nanophotonic structures in case electric-field-insensitive host materials are used [200]. In this way, interference of consecutively emitted photons from the same dopant with an interference contrast of (80 ± 4) % has been achieved, and entanglement between a telecom photon and a long-lived electronic spin has been demonstrated [304]. While this sets the stage for remote-entanglement experiments, further improvements by device engineering will be required toward the up-scaling to large qubit numbers.

#### 6.1.3 QDs at telecom wavelength

With QDs, substantial advances have been achieved in this context at shorter wavelengths [312]. Yet, the reported two-photon interference visibilities of telecom devices are still only moderate. The limiting factor on a microscopic level is noise in the host material and at nanophotonic interfaces, which reduce the carrier phase coherence time and, hence, the photon indistinguishability.

Thus, the *T*_2_ coherence times observed for telecom Stranski–Krastanov InAs/InP QDs grown by MOVPE are in the range of < 200 ps, for example, 74–176 ps) [102], and *T*_2_ = (51 ± 29) ps at saturation power [313]. In the latter report, it was shown that the coherence time is significantly higher, *T*_2_ = (157 ± 72) ps, for droplet epitaxy QDs, which can further improve the characteristics of the InAs/InP material system.

The dephasing of the emitters significantly reduces the interference contrast, e.g., to 14 % for InAs/GaAs QDs grown on a metamorphic buffer layer, even with the resonant QD excitation applied [314]. Other reports on C-band QDs were based either on droplet epitaxy InAs/InP QDs in planar structures [51], [313] or InAs QDs grown on GaAs followed by an InGaAs metamorphic buffer, also located in planar structures [241], [314] or embedded in randomly placed CBGs [220]. For InP-based cavity-coupled QDs with emission wavelengths in the telecom C-band, recent reports show *V* = (19.3 ± 2.6) % for triggered LO-phonon assisted excitation [102], and *V* = (35 ± 3) % using pulsed two-photon resonant excitation of the biexciton–exciton radiative cascade [103].

Two groups have shown recently longer coherence times for QDs grown by MBE with *T*_2_ = (450 ± 20) ps for InAs/InP QD in a tapered nanobeam waveguide [52], and *T*_2_ = (381.4 ± 21.8) ps for InAs/InAlGaAs/GaAs QDs in a CBG cavity [56]. In the latter case, a successful Purcell reduction of the radiative lifetime to *T*_1_ = 257.5 ps combined with long coherence time allowed to achieve high photon indistinguishability at the level of (71.9 ± 0.2) % [56]. Although the QD was excited quasi-resonantly, the relatively high energy difference between the assumed *p*-shell state and the emission energy of 77 meV suggest that the reduced dephasing, originating from optimized MBE growth conditions, and the Purcell-reduced lifetime are crucial for obtaining high TPI visibility.

### 6.2 Approaches to improve the photon indistinguishability

#### 6.2.1 Applying controlled electric fields

Stabilizing the electric field within the devices is key to achieving coherent photon emission from solid-state emitters. A promising approach to this end is to integrate the photon sources into the intrinsic region of a *p-i-n* junction, such that they are sandwiched between highly *p*- and *n*-doped regions, respectively. In this way, carriers can be injected into the QD [39], and their emission wavelength can be tuned via the Stark effect [300]; in addition, also the fine structure splitting in QDs can be tuned [300].

Still, some aspects remain to be shown for devices operating at telecom wavelengths: The removal of excess carriers to lower the charge noise, the control of the exciton charge configuration in QDs, and the electrical tuning of the FSS in a broad range and in a controllable manner.

Charge and spin noise from excess carriers plays the dominant role as a source of spectral diffusion in most nanofabricated environments, although other sources can be relevant as well. This can be reduced by applying an electric bias field [315], [316], [317]. To this end, a vertical *p-i-n* structure can be used, which provides a nonzero and tunable electric field across the intrinsic region. It can remove the excess carriers and accumulate them at interfaces far from the emitters and, as a result, diminish the charge fluctuations. This approach can eliminate the so-called telegraphic noise and lead to reduced emitter linewidths, as observed outside the telecom range with QDs [315], [316], [318] and with NIR color centers in SiC [317], which can substantially improve the indistinguishability [35]. Experiments with erbium dopants and other telecom emitters are an active field of research.

In addition to removing excess carriers, applying a voltage to a *p-i-n* diode with sandwiched QDs allows swiping the confined electron and hole QD states across the quasi-Fermi levels, pinned at the contact, to deplete or inject carriers into the QD confining potential and, consequently, to control the QD charge state. The Coulomb blockade of a gated device was shown to lock the QD charge [319] for short-wavelength quantum rings. Controlling the exciton charge configuration is especially helpful for QDs grown on unintentionally doped wafers, where charged excitons dominate the QD emission. In this way, the probability of neutral complex formation can be increased to maximize the efficiency of polarization-entangled photon-pair generation.

Horizontally defined *p-i-n* structures can also be used to apply an in-plane electric field. This can bring the exciton FSS to zero, demonstrated recently for QDs emitting outside the telecom range [320]. In this experiment, the bottom *n* contact was grounded, and equidistant three top *p* contacts were connected to the mesa containing QDs via bridges. The in-plane electric field at the QD position allows tuning the FSS [320].

For all telecom emitters, the optimal optical resonator geometry for applying an in-plane electric field while minimally distorting the optical cavity mode remains an open question. However, it was shown that electrical contacts can be applied to microrings [321] and required bridges to the CBG resonators [227], [322] with minimal impact on the optical properties. For the latter, the inclusion of bridges is not a fundamental challenge: the electrically contacted devices feature design Purcell factors up to 20, and expected photon extraction efficiency of 70 %–80 %, similar to the unperturbed cavities [227], [322].

#### 6.2.2 Surface passivation

The nanofabrication techniques employed for device fabrication often involve dry etching, which naturally results in defect states at the side walls of the cavity region where the optical field is confined. These defects can form mid-gap trap states that can cause additional energy relaxation channels. This may result in lower photon generation efficiency than the simulated value and can also have detrimental effects on the coherence of the emitted photons.

Therefore, the passivation of cavity sidewalls, aiming at elimination of the surface and subsurface defects induced during the dry etching and subsequent oxidation of the etched sidewalls, is an interesting direction, especially in novel nanostructures with small mode volumes [323]. So far, such passivation was studied with GaAs nanopillars [324] or nanowires [325], as well as InGaAs/InP nanopillars working at telecom [326]. Various solutions were tested, but a dip in ammonium sulfide, (NH_4_)_2_S, followed by encapsulation with a thin layer of SiN [324], or SiO_2_ [326], has emerged as the most common method.

However, recent results with InP-based QWs point out the benefits of encapsulating the active material with a wider bandgap material to maintain the band structure of the device, mitigating etching-induced band-bending effects. Annealing under a phosphine (PH_3_) ambient in an MOVPE chamber decreases the surface recombination velocity by one order of magnitude [327]. This value can be decreased by one more order by regrowing the InP on the structure walls, although it is of limited usage for nanocavities.

With color centers and erbium emitters with tightly bound electrons, an influence of the interfaces to the emitter lifetime has not been observed. Still, passivation of the interfaces is an emerging field of study, with the main goal of increasing the spin and optical coherence and reducing the spectral diffusion linewidth of emitters in nanophotonic devices with small mode volumes.

### 6.3 Optical excitation schemes

**Figure 11:**
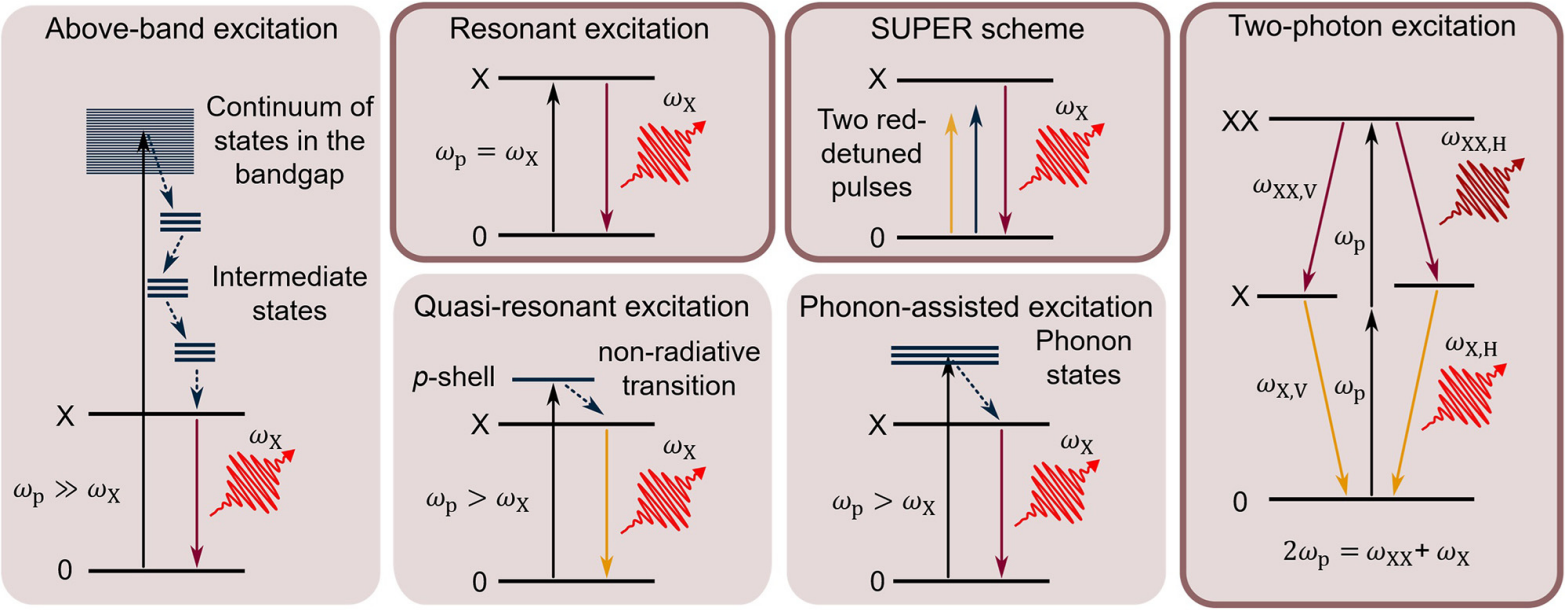
Optical excitation schemes typically applied for the excitation of quantum emitters. Coherent excitation schemes are marked by a framed tile. Left to right: above-band excitation, where the optical pump (*p*) photons have a frequency much larger than the emitting state (X), *ω*_*p*_ ≫ *ω*_X_, and excite the bandgap states of the surrounding matrix material, resulting in an incoherent excitation; Resonant excitation, or resonance fluorescence, where the pump photon energy matches the energy of the probed optical transition, resulting in a strictly coherent driving of the optical transition, *ω*_*p*_ = *ω*_X_; quasi-resonant excitation, where an excited emitter state is pumped, for example, *p*-shell for QDs; schematic illustration and simulation of the swing-up excitation (Swing-Up of Quantum Emitter Population, SUPER), based on protocol introduced in Ref. [239]. In this approach, the application of two red-detuned pulsed coherently drives the two-level system in analogy to resonant excitation but avoids the spectral overlap of the pump and signal photons. Phonon-assisted excitation, where the pump photon energy is relaxed via nonradiative transition, which includes the emission of optical or acoustic phonon, losing the pump coherence, and *ω*_*p*_ > *ω*_X_; two-photon excitation, where the biexciton state is resonantly excited via a nonlinear process, using a virtual state so that 2*ω*_*p*_ = *ω*_XX_ + *ω*_X_. The fine structure of the exciton results in two linearly polarized radiative recombination paths.

Although the electrical injection of charge carriers was used to demonstrate entangled photon generation with QDs, also at elevated temperatures [39], the excess charges negatively impact the device performance with regard to fast and triggered operation, photon coherence, and indistinguishability. In this context, different resonant optical excitation schemes can be used to generate photons from QD devices with improved coherence properties [328]. Some of the most widely used excitation schemes also implemented with QD sources emitting at telecom wavelengths are illustrated in Figure 11: coherent excitation schemes include resonance fluorescence [314], two-photon resonant excitation [41], [103], or the Swing-Up of Quantum Emitter Population – SUPER scheme [245]. A prominent example of incoherent optical excitation is LA-phonon-assisted one [245], [329]. Noteworthy, several other advanced coherent pumping schemes exist, which have yet to be explored and exploited to further improve the quantum optical properties of telecom-wavelength QD quantum light sources. Examples so far only implemented with QDs emitting at shorter wavelengths are adiabatic rapid passage (ARP) in strict-resonant [330] or two-photon resonant [331] excitation using a single chirped laser pulse, or two-photon resonant excitation in combination with a stimulation pulse without [332], [333], [334], [335], [336] and with [337] ARP.

#### 6.3.1 Optical excitation of quantum dots

With QDs at shorter wavelengths, optical excitation has been used to enable choosing the polarization of the emitted photon [333] and switching the emitted photons between pure and mixed states in the photon number basis [336]. Moreover, some protocols require not only the excitation of the QD but also the controlled de-excitation to generate entanglement [338], a concept that was just recently extended for the exploration of high-dimensional entanglement in the photon-number basis [339]. Finally, other schemes are used to create, for example, cluster states using optical pulse sequences [42] or by driving the QD into specific states, which only work with specifically tailored laser pulses [340].

Therefore, optical excitation provides more freedom as one can create pulse sequences of different pulse areas, combine differently detuned pulses, or apply chirp, leading to coherent control of the system, which is impossible when driving electrically. Thus, optical excitation schemes can have many advantages over electrical schemes, but the majority of the protocols are yet to be demonstrated for telecom emitters.

In particular, the application of pulsed resonant fluorescence to QDs is difficult, as it requires sufficient suppression of the excitation laser. Still, the technique is well-developed for devices in the visible, and there is also a first demonstration at telecom [314]. However, also alternative coherent driving approaches that avoid the difficulty of excitation pulse suppression have been implemented. This includes two-photon resonant excitation [41], [103] and the SUPER scheme that has been introduced recently [239] and demonstrated with telecom QDs [245]. It is expected to lead to unprecedented fidelity of the emitter state preparation and thus to an excellent source coherence [239]. So far, however, the experimental results of SUPER and LA-phonon excitation [245] are comparable in terms of multiphoton probability and TPI visibility. Finally, another alternative excitation technique, elastic scattering of excitation laser photons, was demonstrated to lead to coherence times much longer than the Fourier limit with QD devices [51], albeit a trade-off is encountered in the efficiency and in the control of the photon emission time.

#### 6.3.2 Optical excitation of color centers and erbium dopants

Electrical excitation has not been shown with single-emitter devices for erbium dopants and color centers at telecom wavelength. Instead, experiments use pulsed resonant optical excitation. Compared to QDs, this is straightforward because of the longer optical lifetimes; typically, a frequency-stabilized continuous-wave laser and suited modulators are used. For the same reasons, also the excitation laser can be suppressed straightforwardly by temporal filtering, making resonant fluorescence excitation a very common technique.

Optical rather than electrical excitation schemes also have a key advantage: they enable experiments and protocols involving a spin degree of freedom, which can be excited selectively using laser pulses. Thus, the state of this spin can be initialized by optical pumping and read out using resonant fluorescence, as shown with erbium dopants [197], [198] and color centers [176] at telecom wavelengths. In addition, selective optical driving enables the generation of spin-photon entanglement [176], [304], a key technique for quantum networks and repeaters that will be described in Section 7.

### 6.4 Up-scaling to many-emitter devices

Besides improving the emitter coherence and providing suited excitation schemes, there are several other challenges toward building devices that include many single-photon emitters. These will be described in the following.

#### 6.4.1 Emitter localization

To couple many emitters efficiently to their respective optical resonators and thus achieve a reproducible Purcell-enhanced emission, one needs to ensure spectral and spatial matching. However, most of the presented systems so far lacked the required control over the spatial distribution of the emitters. This challenge is particularly relevant in quantum photonic integrated circuits that target the integration of many emitters. Thus, overcoming the inherent randomness in emitter positioning is a key challenge for scalable and deterministic architectures. For color centers and erbium dopants, the preferred choice will likely be to generate the emitters only in the cavity field, as described in Section 4.2.1.

QDs may instead also focus on the localization of emitters, as described in Section 4.2.2, which rely on PL imaging [102], [225]. Difficulties in this technique originate from the high electronic noise level of telecom detector arrays. In addition, it is fundamentally limited by diffraction, and the width of the system point spread function is on the order of the emitter’s wavelength [341], *σ*_diff_ ∼ *λ*_QD_. However, as the Purcell factor strongly depends on the emitter displacement from the center of the cavity on a subwavelength scale, it is desirable to surpass the limits imposed by long-wavelength PL imaging. In this respect, confocal PL scanning is a promising approach. Here, emitters are excited nonresonantly by a focused laser spot while the sample is scanned [250]. The PL emission is then measured in a confocal microscope setup with a high spatial resolution. A significant improvement of the localization accuracy is expected as the point spread function is limited by the laser wavelength reaching down to 10 nm [250].

In summary, both localization and spatially selective emitter generation will likely be improved to required subwavelength precision in the coming years, eliminating the detrimental effects of Purcell factor fluctuations on device performance.

#### 6.4.2 Frequency control of QDs

In addition to spatial control, devices that employ several emitters need to ensure emission at the same frequency. While the narrow inhomogeneous linewidth of erbium dopants and some color centers can easily be bridged by time-resolved detection or low-loss modulators, more effort is needed in the case of epitaxial QDs, where the size and strain distribution causes a much larger inhomogeneous distribution. Thus, *in situ* tuning may be required for functional devices without postselection.

This can be achieved using strain [75], [342], [343] or the quantum-confined Stark effect [312], [318]. Both approaches have the potential to address the challenge of QD ensemble inhomogeneous broadening by fine-tuning the QD energy to match the cavity mode. The Stark effect is typically induced in a *p-i-n* structure, as described above. Here, the electric field can both change the exciton configuration and tune the emission frequency within a charge plateau. A promising route is also a simultaneous application of the quantum-confined Stark effect to tune the emission wavelength and AC Stark effect to reduce the emitter’s FSS, recently demonstrated for QDs emitting outside the telecom range [344].

#### 6.4.3 Frequency control of the optical resonators

Finally, to achieve high source efficiency, the frequency of the resonators needs to be tuned to match that of the emitters. Currently, Fabry–Perot resonators use piezo elements that are bulky and require sophisticated stabilization circuits, hampering their up-scaling. In contrast, nanophotonic devices typically rely on gas condensation tuning at cryogenic temperature. However, this procedure can lack the required long-term stability and may be difficult to implement for many devices on the same chip. Thus, alternative cryogenic tuning approaches will likely be investigated in the coming years to improve the up-scaling potential of chip-based telecom single-photon sources.

## 7 Future directions toward the quantum internet

The realization of a future quantum internet will require breakthroughs in and across various fields, as it requires the implementation of complex multi-user quantum-secured communication networks on the one hand and the development of quantum processing and computing units remotely interfaced via quantum channels on the other hand. In this context, this section discusses possible routes for advancements in the fields of emerging quantum emitter platforms for applications, the realization of quantum communication networks, and quantum computing.

### 7.1 Emerging quantum emitter platforms

While many of the applications discussed in Section 5 have been achieved with QD-based quantum light sources, offering the highest degree of technological maturity to date, there is a plethora of quantum emitter platforms showing prospects for implementations of quantum communication at telecom wavelengths. As discussed in Section 3, this includes sources based on TMDs [92] (Section 3.1.2.6), rare-earth dopants (Section 3.3), and color centers in Si or SiC (Section 3.2). At shorter wavelengths, several emerging 2D materials were considered and evaluated already for their application in QKD, including WSe_2_-based single-photon sources [345], hexagonal boron nitride (hBN) [346] as well as molecules of poly-aromatic hydrocarbons [347], also including an implementation of the B92 protocol using an hBN-based SPS [348]. Given the large variety of degrees of freedom, such as specific material compositions, layer designs, and device functionalities, also the discovery and exploration of novel types of quantum emitter species emitting at telecom wavelengths seems feasible.

### 7.2 Rare-earth dopants

A promising single-emitter platform that has emerged in the last years is rare-earth dopants, as discussed earlier. The photons generated by single erbium emitters have a relatively long duration, typically ranging from ∼1 µs [196], [197] in nanophotonic devices to 100 µs [23] in macroscopic resonators. However, in a typical long-distance scenario, with nodes separated by ≳50 km, the communication rates will instead be limited by the efficiency of the source and by the signaling time, such that the long duration of the photonic wavepacket of erbium emitters is only of minor relevance. In addition, lifetime-limited photon coherence has been demonstrated by avoiding the proximity of interfaces [23] or by using electric-field insensitive integration sites [200]. Together with the long coherence times of the ground state electronic [192] or nuclear spins [193], this opens exciting perspectives for quantum networking with emitters in the telecom C-band.

### 7.3 Quantum communication networks and quantum repeaters

**Figure 12:**
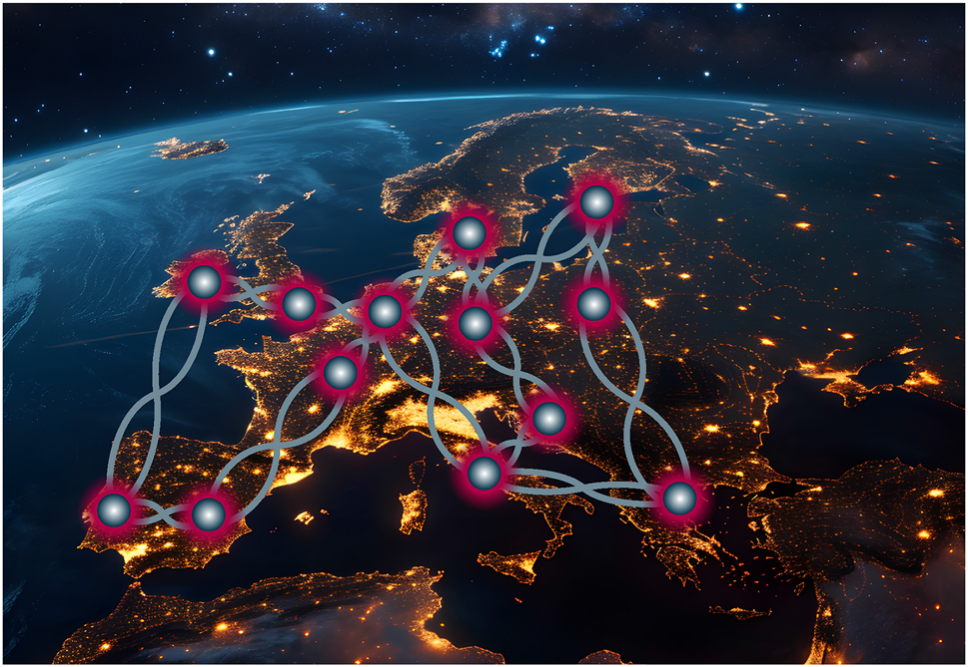
Illustration of a future quantum network on the European continent comprising many long-haul quantum communication links connecting metropolitan areas.

To advance the field of quantum communication beyond the status reviewed in Section 5.1, mainly two research directions need to be pursued: Firstly, it is necessary to implement advanced protocols. In this context, important aspects to be addressed in future work concern the resilience of protocols against device imperfections, the exploration of protocols beyond QKD, and the realization of protocols or architectures overcoming the distance limit of single point-to-point links. Secondly, many quantum-secured links need to be interfaced and managed efficiently in large-scale multi-user networks (see Figure 12).

To achieve resilience against imperfections in physical realizations of QKD protocols, device-independent protocols have been invented. While quantum cryptographic protocols like the BB84 or the E91 protocol themselves provide security at the information theoretical level, their experimental implementations can open loopholes due to device imperfections, which make side-channel attacks possible and hence compromise the protocol’s security (see Ref. [349] for a review on quantum hacking). Fully or semi device-independent (DI) QKD protocols are designed such that imperfections of the devices used for the implementation do not compromise the protocol security, which is a major advantage for practical applications. While fully DI-QKD is extremely challenging to realize, as it requires loophole-free Bell tests [350], also partially device-independent protocols can already be very useful. A prominent example is measurement-device-independent (MDI) QKD [351], [352], for which the protocol security can be guaranteed independently of the measurement device, i.e., the detection setup, which is often the target of quantum hacking. Experimentally implementing such device-independent protocols with deterministic quantum light sources, substantial advantages could become possible compared to previous realizations using faint laser pulses [353], [354], [355].

The field of quantum cryptography, however, is not limited to QKD by far, and new cryptographic functionalities must be explored in future work. Indeed, there are many important cryptographic tasks that require the implementation of cryptographic primitives in distrustful settings, which are not covered by QKD [356]. Examples are two- and multi-party cryptographic primitives such as coin flipping, bit commitment, or oblivious transfer, which are building blocks for many sensitive tasks in modern communication networks, including secure authentication. While these advanced cryptographic tasks might benefit substantially from the use of on-demand single- and entangled-photon sources, none of these have been implemented so far with sub-Poissonian quantum light sources, neither at short nor at telecom wavelengths. Future work in this direction may open up an entirely new field of research.

As the communication distance in direct point-to-point QKD is limited by photon loss, both in free space and in fiber-optical links, advanced architectures are required for long-distance scenarios. In principle, intermediate trusted nodes can be introduced, as impressively demonstrated in the largest quantum communication network to date, spanning 4,600 km between the Chinese cities of Beijing and Shanghai [357]. The intermediate trusted nodes, however, reduce the overall security level. The ultimate solution to cover arbitrary distances without compromising security is quantum repeaters.

The first proposal of quantum repeater schemes by Briegel, Dür, Cirac, and Zoller in 1998 overcomes the distance limit by using entangled photon pair sources, entanglement swapping, and quantum memories as quantum resources [358], [359]. While different solid-state quantum emitter platforms are available and considered for their implementation in quantum repeater schemes [360], the compatibility to quantum memories remains a challenge in itself [361]. Recently, however, the deterministic storage and retrieval of telecom C-band photons from a QD single-photon source in a quantum memory was reported [362]. Using a high-bandwidth quantum memory based on an ensemble of rubidium atoms, a total internal memory efficiency of (12.9 ± 0.4) % was achieved – an advance showing prospects for applications in quantum repeater networks. Still, further improvements are required, especially in the bandwidth matching between the quantum memory and the quantum emitter.

As an alternative to memory-based quantum repeaters, all-photonic measurement-based quantum repeater schemes are considered [363], [364], [365], [366]. Photonic cluster states, as required for this type of repeater, have already been generated at shorter wavelengths using QD-based quantum light sources [42], [367], [368], and the feasibility of implementing similar experiments with color centers in diamond was evaluated very recently [369]. Transferring this knowledge to telecom wavelengths in future work will pave the way to substantially reduce transmission losses for all-photonic quantum repeaters. This, however, may also require the identification of protocols with a much higher resilience against imperfections or substantial further improvements in device performance.

Having realized different types of individual quantum-secured communication links, finally, the interconnection to larger networks must be addressed. A future quantum network will most likely not be constituted of a single technology or a specific protocol, as these are specific to the communication scenario and use case. Assuring end-to-end security when interfacing different types of protocols or cryptographic primitives, which is known as composability in information theory, is a major challenge in this context. In addition, the quantum physical and quantum logical layers must be interfaced and designed such that a seamless integration in application layers of end-users is possible [357].

### 7.4 Quantum computing

In the field of quantum computing, one can distinguish approaches aiming at full-fledged quantum-error-corrected quantum computers, representing the final stage of quantum information processing, or less complex machines providing a quantum advantage for very specific computational tasks. As resources, on the other hand, flying or stationary qubits can be used. More specifically, in the context of solid-state quantum emitters, either photonic states or spin qubits can be used for the processing of quantum information.

A concept that can solve specific problems, such as simulating molecular vibronic spectra [370], much faster than classical computers is boson sampling, as introduced by Aaronson and Arkhipov [371]. In boson sampling, the probability distribution of scattered indistinguishable photons is modeled in a linear interferometer [372] requiring much less quantum resources than universal quantum computers. First experimental realizations of boson sampling using QD-based quantum light sources emitting around 900 nm [311], [373], [374], [375] have demonstrated the advantage of deterministic quantum light sources as compared to probabilistic sources in terms of scalability. To transfer this knowledge to telecom wavelengths and further improve the performance of boson sampling machines, substantial advances in the scalable generation of photons with high two-photon interference visibility will be required. For the latter, spatially demultiplexing GHz-clocked single-photon sources appears currently to be the most feasible route compared to using multiple indistinguishable sources.

Universal quantum computation schemes based on flying qubits date back to 2001 with the proposals for linear optical quantum computing [376] and measurement-based, or one-way, quantum computing [377]. Both schemes are based on quantum interference and measurement-induced nonlinearities without direct photon–photon interactions and have been developed further [378]. A quantum resource essential for so-called one-way quantum computers are photonic cluster states (cf. all-photonic quantum repeaters above), produced, for instance, by the repeated generation of spin-photon entanglement [368], [377], [379]. Producing such states with high efficiency will allow the use of quantum gates with a high success rate and a sufficient signal-to-noise ratio for quantum processing. While for QD-generated single-photon states at 1,550 nm the observed detector click rates already exceed 10 MHz [220], the proof-of-principle generation of even simple cluster states remains an important next hurdle to overcome.

Moreover, quantum memories are an important building block in photonic quantum computing, enabling quantum teleportation between flying and stationary qubits [307] as well as quantum error correction [380]. Spin coherence times can exceed 100 µs for QD-confined electrons [381] and tens of minutes up to several hours for ionized donors in silicon [382] at room and cryogenic temperatures, respectively. Still, implementing architectures with solid-state quantum memories requires efficient spin-photon interfaces for the storage and retrieval of quantum information. At telecom wavelength, besides erbium dopants also atomic vapors [361] and Nd^3+^-doped crystals [383] are investigated, as discussed earlier [362].

Another fundamental building block in quantum computing is quantum logic gates, which can be realized using either flying qubits from quantum emitters [312] or spin qubits in the solid state [163], [175], [384]. Implementing such schemes experimentally with quantum light sources at telecom wavelength remains an open challenge.

Today the best-performing quantum light sources and spin qubits, which also serve as quantum memories, are realized in different platforms. Ultimately, it is worth striving for core technologies enabling both scalable fault-tolerant quantum networks and computers, for which the erbium- and color-center platforms show prospects.

Toward the implementation of quantum networking using erbium emitters, the next key step is the entanglement of remote spin qubits via photon interference or spin-photon quantum gates [175]. This further requires optical single-shot readout of the electronic spin, as demonstrated in several materials [176], [197], [200], [204], together with microwave pulses to control the spin and decouple it from interactions. In addition, the emission frequency of the photons needs to be stable during the entanglement protocol.

These requirements have been achieved with erbium dopants [23], [200]. In this case, tailored rephasing protocols [25], [304] can overcome the need for large degrees of photon indistinguishability and enable remote entanglement even for emitters with considerable spectral diffusion, as observed in current devices. With these advances, the entanglement of remote erbium dopants will likely be achieved in the near future.

Telecom color centers offer many prospects in this context as well. Both silicon and silicon carbide can host long-lived spins serving as quantum memories with long coherence times [382] and high fidelity [385]. Photonic connectivity is enabled at telecom wavelengths by several emitters, including T-centers in silicon [168] and transition metal color centers in SiC [158], [161]. Both platforms allow for scalable fabrication of integrated photonic devices, as demonstrated recently with erbium dopants [203], see also Section 4.1.2. Envisioned applications include memory-assisted MDI-QKD [386], distributed quantum computing, blind quantum computing [387], and enhanced sensing – applications that, once successfully implemented on a single platform, would pave the way for scalable quantum information processing [163].

## 8 Conclusions

In this review, we have summarized the current state of solid-state quantum emitters operating in the telecom wavelength range. The covered systems include epitaxial QDs and QDs in 2D materials, color centers, and erbium dopants. We discussed epitaxial methods for the QD growth and ion implantation to create color centers and introduce dopants to the solid-state host crystal. Although the described systems differ in their fabrication method and some of their characteristics, they share many features and challenges as a result of their placement in a crystalline host material.

We have provided an overview of photonic devices based on the discussed quantum emitters, highlighting both the challenges and strategies for effective implementation. Furthermore, we included a comparative analysis of the key parameters, such as photon extraction efficiency, Purcell enhancement, lifetime, suppression of multiphoton emission, and photon indistinguishability. We further summarized recent applications of solid-state quantum light sources operating in the telecom wavelength range, focusing on key areas such as quantum key distribution, entanglement distribution, and quantum teleportation. Additionally, we have addressed the current challenges and limitations in the field, including scalability, photon coherence, and indistinguishability. Finally, we have outlined our vision and future directions in the field of photonic quantum information technology.
